# A new ensemble heart attack diagnosis (EHAD) model using artificial intelligence techniques

**DOI:** 10.1038/s41598-025-18129-0

**Published:** 2025-09-15

**Authors:** Bahaa El-Din Waleed, El-Sayed M. El-Kenawy, Sherif Ibrahim, Hossam El-Din Moustafa, Asmaa H. Rabie

**Affiliations:** 1 Department of Applied Health Sciences, Higher Technological Institute of Applied Health Sciences, Mansoura, Egypt; 2 Department of Communications and Electronics, Delta Higher Institute of Engineering and Technology, Mansoura, 35111 Egypt; 3 Faculty of Medicine, Mansoura University, Mansoura, Egypt; 4 Department of Communications and Electronics, Faculty of Engineering, Mansoura University, Mansoura, Egypt; 5 Department of Computers and Control Systems Engineering, Faculty of Engineering, Mansoura University, Mansoura, Egypt; 6 Applied Science Research Center, Applied Science Private University, Amman, 11931 Jordan; 7 Dean of Faculty of Artificial Intelligence and information, Horus University, New Damietta, Egypt; 8 Computer Science Department, Arab East Colleges, Riyadh, Saudi Arabia

**Keywords:** Diagnosis system, Feature selection, Heart attack, Artificial intelligence, Ensemble model, Medical research, Experimental models of disease

## Abstract

Myocardial infarctions, also known as heart attacks, are a leading cause of death globally, highlighting the need for prompt and precise diagnoses to improve patient outcomes. Recently, many machine learning (ML) and artificial intelligence (AI) techniques have been used for diagnosing heart attack diseases, but these techniques still cannot provide the most accurate results. Thus, it is important to improve these approaches to provide better results than current methods do. In this paper, a new hybrid diagnostic approach for heart attack diagnosis called the ensemble heart attack diagnosis model (EHAD), which is based on the ensemble classification technique (ECT), is introduced. ECT integrates three primary classifiers, namely, the support vector machine (SVM), long short-term memory (LSTM), and artificial neural network (ANN), which are combined with the majority voting (MV) technique to make accurate and fast final decisions. The simulation proved that the Ensemble Heart Attack Diagnosis (EHAD) outperforms other models related to many metrics, such as recall, precision, F1 score, accuracy, and many other statistical analysis measurements.

## Introduction

Myocardial infarction, another name for heart attack, is a major cause of death globally. For prompt medical intervention and better patient outcomes, heart attacks must be detected early and accurately. ML methods have demonstrated significant promise in helping medical practitioners identify and categorize heart attacks early, allowing for individualized patient treatment regimens. ML has the potential to completely transform cardiovascular treatment by offering precise and automated categorization models for heart attacks, especially in light of the increasing accessibility of electronic health records and data science breakthroughs. ML algorithms provide promising prospects for precise heart attack classification, early diagnosis and customized care. Despite these challenges, owing to developments in this area of study, enormous improvements have been made to enhance patient outcomes and decrease the financial cost that heart disease places on the medical system^1^.

Statistical models and rule-based systems have been the backbone of traditional cardiac disease detection techniques; however, these techniques are not always equipped to handle intricate, high-dimensional data. With the need to manually select features and learn directly from patient data, developments in AI and ML provide a revolutionary chance to increase diagnostic efficiency and accuracy. Recent applications of AI, such as deep learning (DL), permit easy access to large medical databases in real time, giving rise to timely forecasts and insights that guide preventive care and improve patient outcomes^2,3^. We need AI-enhanced diagnostic systems to address complicated clinical cases and improve patient care systems with effective resources and treatment allocation in the wake of the world-heart disease boom^4^.

The improvements in AI and optimization have made it possible to increase the number of heart disease predictive models. Optimization algorithms such as particle swarm optimization (PSO) and the genetic algorithm (GA)^5–7^ are combined with AI models such as convolutional neural networks (CNNs), ANNs and SVMs to fine-tune more accurate estimates obtained from these models to assist physicians in identifying patients at greater risk of illness. The implementation of such techniques is crucial to dependable medical diagnoses since their combination with optimization procedures has significantly approached essential issues such as overfitting and interpretability, according to the research in^8,9^.

This work introduces a major contribution of a diagnostic model named the ensemble heart attack diagnosis (EHAD) model for the diagnosis of heart attack. Its primary purpose is to speed up and guarantee precise results in diagnosing heart attacks. The model includes an Ensemble Classification Technique (ECT), which utilizes the positive aspects of three major classifiers: support vector machine (SVM), artificial neural network (ANN), and long short-term memory (LSTM). The use of a majority voting (MV) mechanism over the individual outputs of all classifiers enables the ensemble heart attack diagnosis (EHAD) model to produce an accurate final diagnosis. It is shown via simulation that the current approaches are outperformed by the Ensemble Heart Attack Diagnosis (EHAD) model on a variety of critical assessment dimensions, such as accuracy, precision, recall, F1 score, and numerous statistical analysis measures. The structure of this paper is as follows: the literature review is discussed in section two, and section three reviews the proposed ensemble heart attack, diagnosis (EHAD) model. In section four, the methods used are described. The simulation and results are discussed in section five, and finally, the conclusion is presented in section six.

## Related work

In this section, we discuss the literature review^10^. Although ensemble models, including support vector machine (SVM), k-nearest neighbors (KNN), logistic regression (LR), random forest (RF), naive Bayes (NB), decision tree (DT), and extreme gradient boosting (XGBoost), have been shown to perform well in terms of diagnosing heart disease^11^, some issues are crucial. The metaheuristic methods employed to carry out the process of feature selection, such as the gray wolf optimizer (GWO) and the Pearson correlation coefficient (PCC), increase the power of the model to recognize informative trends out of the complexity of medical data. The combinations are able to limit the weaknesses of individual models, maximize generalization and feature relevance, and minimize overfitting, thereby enhancing diagnostic precision. Nevertheless, in addition to these strengths, several limitations should be mentioned. First, the interpretability of models increases when models become increasingly complex via ensemble approaches. In clinical practice, black-box models can slow physician trust and acceptance, particularly when critical decisions are made accordingly. Medical practitioners are always in need of a clear explanation of predictions, and ensemble methods do not always provide such explanations. Second, training and tuning multiple classifiers and optimizers is costly. This is even more compounded by the fact that in the case of real-time (or resource-limited) cases (e.g., mobile health apps, remote clinics), the situation becomes quite complex. The process of optimizing ensemble pipelines usually demands sophisticated hardware or competence, which presents scalability concerns. Third, the ensemble methods can potently enhance the diagnostic performance but at the same time endanger overfitting in the case of misconfigured methods, particularly datasets including noise or those of poor size. Even with techniques such as GWO that help prevent overfitting, they also add new hyperparameters and stochastic behavior, which in all cases might not be very stable when switching between datasets. Furthermore, the ability of feature selection through PCC and GWO is only effective in capturing nonlinear or interactive correlations between features and is not very possible in situations unless rigorously intertwined with domain knowledge or nonlinear feature finders. This may cause unsuspected otherwise clinically significant patterns to be excluded. Finally, although ensemble learning at least has the potential to perform well as a prediction tool, there are certain ethical aspects of it and data governance to consider, particularly with respect to sensitive patient data. The risks and trade-offs among performance, privacy, and interpretability should be considered to achieve the responsible use of AI in healthcare practices. To conclude, although ensemble methods present an effective solution that can increase the level of accuracy of diagnostic decisions, the practical implementation of ensemble methods in clinical conditions should be associated with interpretability, complexity, and computational trade-offs. Future work should focus on interpretable ensembles, trainable pipelines and clinical applicability to make them better fit clinics and to improve overall trust (Table 1).

**Table 1:** Many recent medical diagnostic models.

Paper	AI Techniques	Optimization/Feature Selection	Accuracy	Advantages	Disadvantages
Ensemble Heuristic–Metaheuristic Feature Fusion Learning [11]	SVM, KNN, LR, RF, NB, DT, XGBoost	GWO, PCC	Cleveland: 91.8% Statlog: 88.9%	High diagnostic accuracy, efficient feature selection	Computational complexity, longer training time
Prediction of Heart Disease with Jellyfish Optimization [12]	SVM	Jellyfish optimization	98.47%	Low-cost, high sensitivity and specificity	Requires expertise, limited interpretability
HypGB Classifier [13]	Gradient Boosting	HyperOpt, LASSO	Cleveland: 97.32% Kaggle: 97.72%	High accuracy, optimized feature selection	Complexity, data dependency
Hybrid ML with Deep Learning and Meta-Heuristic Algorithms [14]	CNN, SVM, Autoencoder	PSO	COVID-19: 99.76% Brain tumor: 99.51%	High accuracy, dimensionality reduction	Complexity, interpretability
TANFIS Classifier [15]	TANFIS	Moth Flame, Grasshopper Optimization	99.76%	High efficiency, IoT integration	High computational overhead, interpretability challenges
Naive Bayes with Feature Selection [16]	Naive Bayes	Backward Elimination	94.89%	Simplicity, ease of interpretability	Assumption of feature independence, less expressive
Wavelet Denoising with ANN [17]	ANN	Wavelet Denoising	97.05	Improved noise reduction in ECG signals	Sensitive to SNR changes, tuning complexity

The study conducted in^12^ revealed the possibility of enhancing the prediction of cardiac diseases via the combination of support vector machine (SVM) and other machine learning (ML) methods for feature selection, especially when the Jellyfish optimization algorithm (JOA) is used. Although this method contributes best to ML in regard to affordable and evidence-based diagnostics, several limitations must nevertheless be considered. First, decreased overfitting and model generalizability are possible when the JOA is used to determine features, but the interpretation of these combinations of learning technologies becomes more troublesome. In many cases, clinicians demand explainable and understandable processes of decision-making, but numerous ML-based frameworks, particularly those based on metaheuristic optimization, are black boxes, which creates a challenge to justify a diagnosis or develop clinical confidence. Second, expertise within a field is critical in the implementation of these techniques. With the help of automated feature selection, the effective application of SVM-JOA frameworks requires professionally trained specialists who are able to preprocess data correctly, optimize hyperparameters, and interpret results correctly. This dependence can cause issues in the availability within the lean environment or institutions with a shortage of technical personnel. Furthermore, there is a concern of model generalizability. The data heterogeneity, the shift in its distribution, and the selection biases may cause the ML models prepared on certain datasets to not work as expected when applied to other populations. The inability to validate the model in different cohorts decreases the reliability and applicability of the model in clinical practice. Finally, although the studied increased performance scores are encouraging, only a few papers have compared the trade-offs between accuracy and computational cost. Metaheuristics, such as JOA, may be intense and computationally expensive and can take some time; thus, the actual practical use of such procedures in clinical workplaces may be questioned. To draw a conclusion, SVM with JOA introduces a new potentially strong method for predicting heart diseases, but it has to be investigated in the future to be more explainable, transferable across datasets, and understandable whether it is easy to utilize in medical practice.

Although the experiment conducted in^13^ provides evidence of the benefits of highly efficient advanced AI techniques such as gradient boosting (GB) and LASSO in detecting cardiovascular disease correctly, there are several important obstacles to the applicability of the study in real-life scenarios. The ability of LASSO to conduct automatic feature selection increases interpretability and efficiency since irrelevant clinical variables are filtered out. Combined with HyperOpt to optimize the hyperparameters of the GB, the model yields high amounts of precision. Nevertheless, this degree of performance can be determined by the characteristics of the dataset, i.e., insufficient diversity or imbalance, which can interfere with its generalizability to larger populations. In addition, although GBs have an ensemble property that lowers the variance and enhances robustness, their black-box observations can decrease clinical interpretability, which is important in high-stakes medical environments. Medical workers are frequently in need of simple and understandable knowledge that proves that something must have been chosen and applied. Additionally, although computational frameworks such as HyperOpt are more effective at tuning the model, they are more complex and computationally expensive; thus, they are more unattainable in low-resource settings, including small clinics or rural health stations. Another fear is data dependency: a model that has been fine-tuned with data from one clinical population may not work equally effectively with data from other demographic or regional populations because of differences in genetic, lifestyle, or socioeconomic factors. This is why careful cross-validation, domain adaptation, and clinician-in-loop approaches must be developed to decrease the gap between research performance and clinical applicability.

The research in^14^ proposed a hybrid model of diagnosis that uses a combination of AI approaches such as particle swarm optimization (PSO), autoencoders, and convolutional neural networks (CNNs) in classifying medical data. The use of such integrative methodology is laudable, as it enables optimization and deep feature extraction, which can enable better classification performance as well as robustness in handling multifaceted and high-dimensional clinical data. Nevertheless, the model has serious drawbacks in addition to the advantages of its performance since it is highly limited in terms of interpretation and transparency. Other deep learning architectures, such as autoencoders and CNNs, can be treated as black boxes, and sometimes, clinicians can hardly know the reasons behind certain predictions or justify them. Such explainability may negatively impact trust and clinical adoption, particularly in important areas such as cardiovascular diagnostics, where explainable AI (XAI) is becoming a requirement. Additionally, optimization-based models such as PSO might have to be highly customized and time-resource-consuming, increasing doubts regarding scalability and deployment in practical hospital environments. Additionally, the hybrid method can be problematic regarding generalizability to alternative data or other populations of patients unless trained with diverse and representative information. The model may not be universal unless it is validated in different institutions or among different demographic populations. As a result, although the application of PSO and deep learning shows promise, the potential in future research is to focus on making the model explainable, limiting the computational load, and testing the model in a variety of real-life situations to make it relevant in different clinical scenarios.

In accordance with^15^, a sophisticated hybrid diagnostic system based on the principles of the grasshopper optimization algorithm (GOA) was suggested to identify heart disease. The method incorporates swarm intelligence into a machine learning framework that improves the accuracy of diagnoses and their versatility. The collection and monitoring of data in real time with the help of fog computing and the Internet of Things (IoT) is an interesting development that is expected to solve the problems of latency and speed of response in clinical situations. However, despite good performance, the method has considerable limitations. First, hybrid AI models are commonly transparent, also known as the black box issue, which is an obstacle to clinical belief and display. Second, the complexity of algorithms such as GOA or moth-flame optimization (MFO) may require extensive resource usage, which rules out their implementation in resource-limited applications or real-time implementations. Furthermore, the arrangement of these systems may involve numerous parameters (e.g., swarm dynamics, fitness landscape, and neural network weights), which need to be tuned to achieve good performance on a specific dataset and render such systems challenging to work effectively or morph effectively on different datasets. Although simpler implementations, such as logistic regression or decision trees, might have slightly worse performance, they will still have more explainability and compatibility with including them in clinical workflows. This trade-off of performance and interpretability continues to be one of the key issues when attempting to translate hybrid AI systems into reliable, real-world medical technologies.

Although the hybrid diagnostic model suggested in^16^, which combines the feature selection approach with the naive Bayes (NB) method, seems to be rather simple and easy to interpret and is thus often required in the clinical setting, it is crucial to draw critical attention to the assumptions and limitations of applying this model to real datasets that consider heart disease. Naive Bayes is based on the idea of conditional independence of features with respect to the class label. However, this is rare in medical data where clinical and physiological variables (blood pressure, cholesterol levels, ECG results, etc.) tend to have a complicated multiplicity of interdependencies. Failure to fulfill this assumption may result in biased estimation of probability and, consequently, poor performance of the typicality of a model. Additionally, although the model uses backward feature elimination to substantiate a feature subset and optimize the classification performance, the elimination process may likewise filter all information-generating weak features without realizing that weak individual features may have a collective meaning. This might minimize the robustness of the model and cause the model to discard valuable diagnostic information. The simplicity of NB, although attractive in computational terms, limits its use in modeling more complex, nonlinear relationships that are evident in heterogeneous populations of patients. This shortcoming is even more keenly felt when one applies it to high-dimensional data or when feature interactions are vital to class distinction, as it is able to distinguish between the first and second stages of heart disease as opposed to the later stages. The scalability of NBs is another important pitfall of this technology. It is relatively fast but appropriate for small datasets; it works well, but the performance can be low when the dataset contains a tremendous number of observations or when the variables are heavily multicollinear. Larger feature interactions and adaptability to imbalanced data are the main characteristics that make models such as random forests, gradient boosting machines, or deep learning architectures far more predictive in such cases. Additionally, looking at the issue of deployment, it may not suffice to make use of a model that is comparatively lower in capacity, such as naive Bayes, to make use of real-time systems of clinical decision support where sensitivity and specificity are essential. Its interpretability is a plus but frequently incurred at the expense of loss of diagnostic strength. Therefore, although the NB-based hybrid model can potentially be used as a baseline or a preliminary tool, it is rather unsuitable in large-scale and high-stakes cardiovascular diagnosis environments unless it is either complemented by more severe machine learning methods or implemented in a greater ensemble construct.

In medical studies, according to^17^, one can detect heart conditions through the integration of artificial neural networks (ANNs) with wavelet techniques for denoising, especially in terms of accuracy when the ECG signals have some noise. Wavelet denoising is instrumental in removing signal distortions and interference with the input data fed into the ANN through enhancing the quality of the input data. This improved quality of data can greatly increase the accuracy of its classification, particularly in real-time ECG monitoring systems. Additionally, the fact that ANNs can be used to model nonlinear relationships enables them to detect and classify complex cardiac patterns and thus present superiority to more traditional rule-based systems. However, several obstacles restrict the popularity and strength of such an approach. First, the tuning of wavelet denoising parameters, which include the type of wavelet to be used, the level of decomposition, and the thresholding style, is generally not trivial and very prone to trial and error. This limits the accessibility of the method to nonexpert users and may limit the reproducibility of the techniques in different studies. In addition, ANN models, even though they are very powerful, are vulnerable to training data quality and quantity. The use of poor-quality annotations or data imbalance may cause overfitting or worse performance, which reduces model generalizability in clinical practice. The other crucial limitation is that ECG signals may differ greatly among different individuals because of the physiological variation, presence of another condition, and noise in the sensor, and such variance may not be addressed by the flexibility of a pretrained ANN. The model may not always have an attitude in ensuring that different patient populations have a high degree of accuracy unless it has enough personalization or adaptive learning techniques to assure that. Moreover, ANN models regularly perform as black boxes, and a clinician can hardly understand the principles of making certain judgments, which prompts questions about transparency and reliability in medical diagnosis. In general, the combination of wavelet denoising with an ANN has the potential to increase the accuracy of heart disease diagnosis when an ECG is used; however, several essential challenges in terms of explanation, parameter setting and generalizability need to be overcome before wavelet denoising may be deployed in practice whenever such techniques are applied in the health care environment.

## The proposed ensemble heart attack diagnosis (EHAD) model

**Fig. 1:**
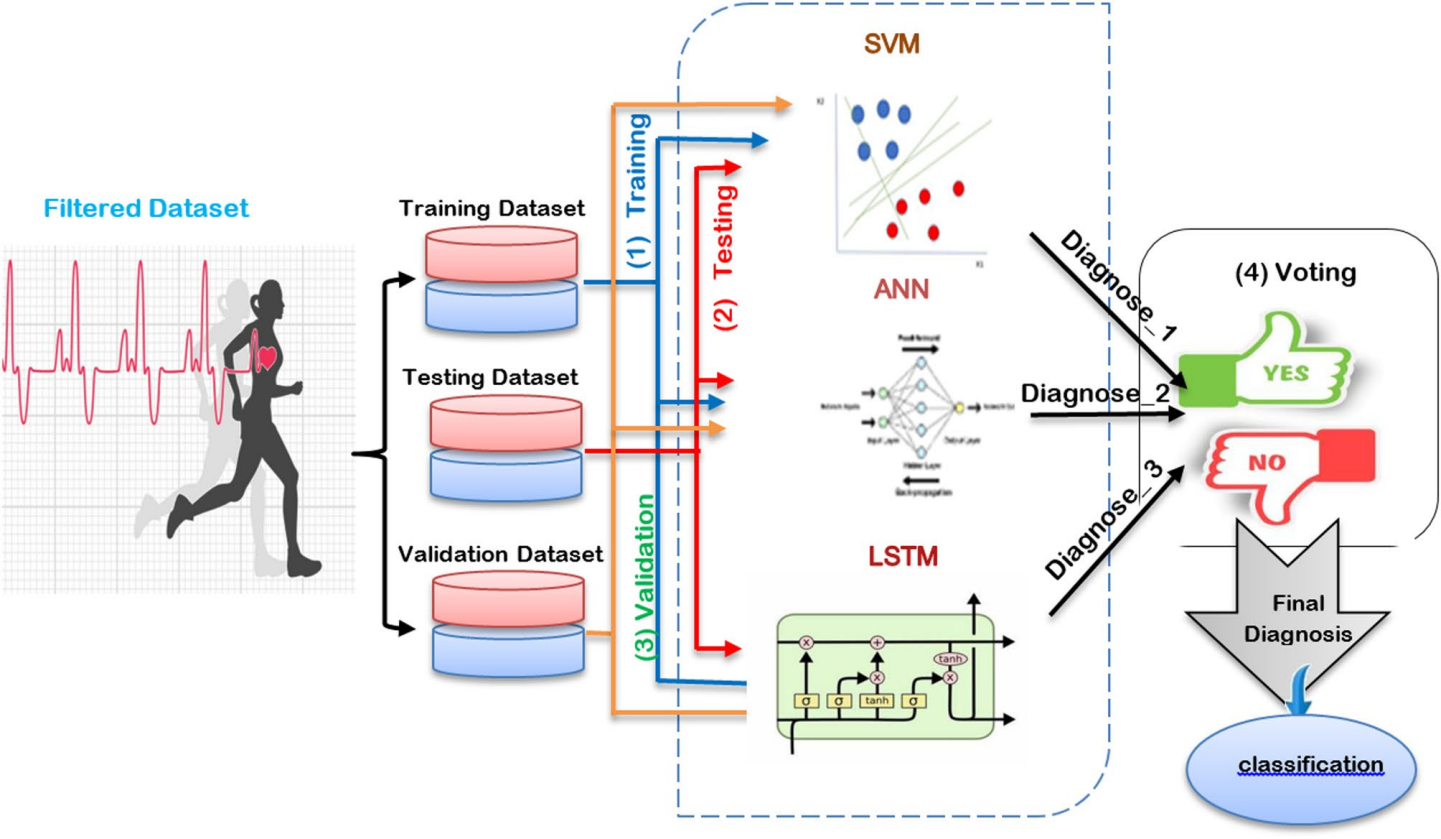
The Structure of the Ensemble Heart Attack Diagnosis (EHAD) Model.

In this section, an ensemble heart attack diagnosis (EHAD) model that uses the ensemble classification technique (ECT) is proposed as a new hybrid diagnostic approach for heart attack diagnosis. ECT integrates three primary classifiers, namely, SVM^18^, LSTM^19^, and ANN^20^, which are combined via the majority voting (MV) technique to make a final decision, as illustrated in Fig. 1. SVM is a boundary-based classifier, LSTM is a sequential learning technique, and ANN is a flexible neural network approach. Initially, these three techniques are trained on the same dataset in parallel. Next, they are tested on the same testing dataset in parallel. After testing, these techniques are validated in parallel to provide their individual decisions. Finally, the diagnosis with the most votes is selected via the MV technique, which gathers the decisions from the SVM, LSTM, and ANN to deliver an accurate final decision for heart attack prediction. To implement ECT, four steps must be followed, as shown in Fig. 1. In the 1 st step, the SVM, LSTM, and ANN are trained; in the 2nd step, these techniques are tested; in the 3rd step, they are validated; and in the 4th and final steps, the accurate final decision is determined by the MV technique on the basis of the decisions of the SVM, LSTM, and ANN.

The proposed EHAD model introduces a new level of increase over the existing methods because it takes a wide range of classifiers, including SVM, ANN, and LSTM, to make a majority vote ensemble. The combination of SVM and ANN in this ensemble has the advantage of utilizing the strengths of each in different dimensions, e.g., SVM in high-dimensional spaces, ANN in learning nonlinear relations, and LSTM in time-based relation learning in clinical datasets. Moreover, although in previous models, it was common to prioritize selecting features by applying metaheuristic algorithms, such as GWO or the JOA, the EHAD framework maximizes generalization with SMOTE-based oversampling, which is more helpful in contending with groups that are underrepresented. Another important difference is the ensemble method: other methods use the computationally expensive and usually black box method of ensembles with weights, whereas in EHAD, a majority voting process is used, which is configured to achieve high recall of the LSTM; this is essential to prevent false negatives in medical diagnosis. This leads to a model that can be proven to be competitive in terms of all three indicators of accuracy, precision, and recall without artificial boosting of the indicators. In addition, the EHAD ensemble appears to be computationally less expensive and more interpretable than hybrid deep learning (e.g., PSO-CNN or GOA-based pipelines), thereby making it more applicable to a clinical setup when deployed as a real-time system. Through the balance of impactful performance, interpretability, and scalability, EHAD is a viable, trustworthy, and medically consistent discovery in the translation of artificial intelligence to diagnose heart diseases.

The reason for using these techniques is that EHAD combines the three methods SVM, ANN, and LSTM because the performance of each technique can overcome the challenges of other techniques. Each technique offers distinct advantages for diagnostic and predictive tasks, and a comparison of the three methods is provided in Table 2. SVM is better with small datasets, which provides effective performance due to its support vectors and margin maximization and is more robust to overfitting with a regularization parameter^21,22^. SVMs also have better interpretability than do ANNs, which tend to overfit without additional regularization methods^23^. Although ANNs are better for handling large datasets, they can learn nonlinear patterns automatically and are versatile over various tasks^24^. While LSTM is better than SVM in time series tasks and sequential tasks, SVM is more useful in dealing with nonsequential data^25^. ANNs are easier to train and more versatile for nonsequential tasks, whereas long short-term memory (LSTM) is better than ANNs in tasks requiring a sequence of modeling and long-term memory retention^26–28^.

**Table 2:** Comparison between the three methods (ANN, SVM, LSTM) used in EHAD.

Aspects	ANN vs.SVM	LSTM vs. SVM	ANN vs. LSTM
Data size	SVM excels in small dataset. ANN needs large datasets.	SVM is better for small datasets. LSTM needs more data.	ANN performs well on non- Sequential data. LSTM excels in time-series.
Sequential Data	ANN struggles. SVM also requires feature engineering.	SVM cannot handle sequential data. LSTM is designed for it.	LSTM excels with sequences. ANN cannot handle them Inherently.
Feature Learning	ANN learns features automatically. SVM relies on manual feature.	SVM requires manual features. LSTM learns temporal features.	LSTM captures long-term Dependencies. ANN cannot without added Complexity.
Nonlinearity	ANN handles complex patterns.	LSTM handles sequential non Linearity better.	ANN is simpler. LSTM handles temporal non Linearity better.
Computational Efficiency	SVM is efficient for small datasets.	SVM is computationally lighter for Non sequential data.	ANN is faster and simpler to Train for nonsequential tasks.
Interpretability	SVM has clearer decision boundaries	SVM is more interpretable. LSTM is a black box.	ANN is simpler but still a black box compared to interpretable SVM

## Methods

This research uses three different but complementary machine learning methods and deep learning methods to create a strong and hybrid diagnostic model for predicting heart attack: SVM, ANN, and LSTM. They have their own advantages in addressing various complexities of data. SVM is an efficient supervised learning method that performs well in binary classification problems, especially when decision boundaries between classes are well defined^21,22^. Many of these methods are useful for high-dimensional medical data because of the possibility of finding the optimum separating hyperplane and the use of kernel functions. An ANN, in turn, is a dynamic and very versatile model that can interpret nonlinear relationships in patient data, which qualifies it to identify intricate patterns among distinct clinical attributes^29,30^. LSTM is a specialized format of a recurrent neural network (RNN) aimed at working with so-called sequential dependency and time series information, which is crucial in the segment of medical diagnostics when how a set of symptoms or values of various tests change over time is important^31,32^. The details of SVM, ANN, and LSTM are described in the next subsections.

### Support vector machine (SVM)

**Fig. 2:**
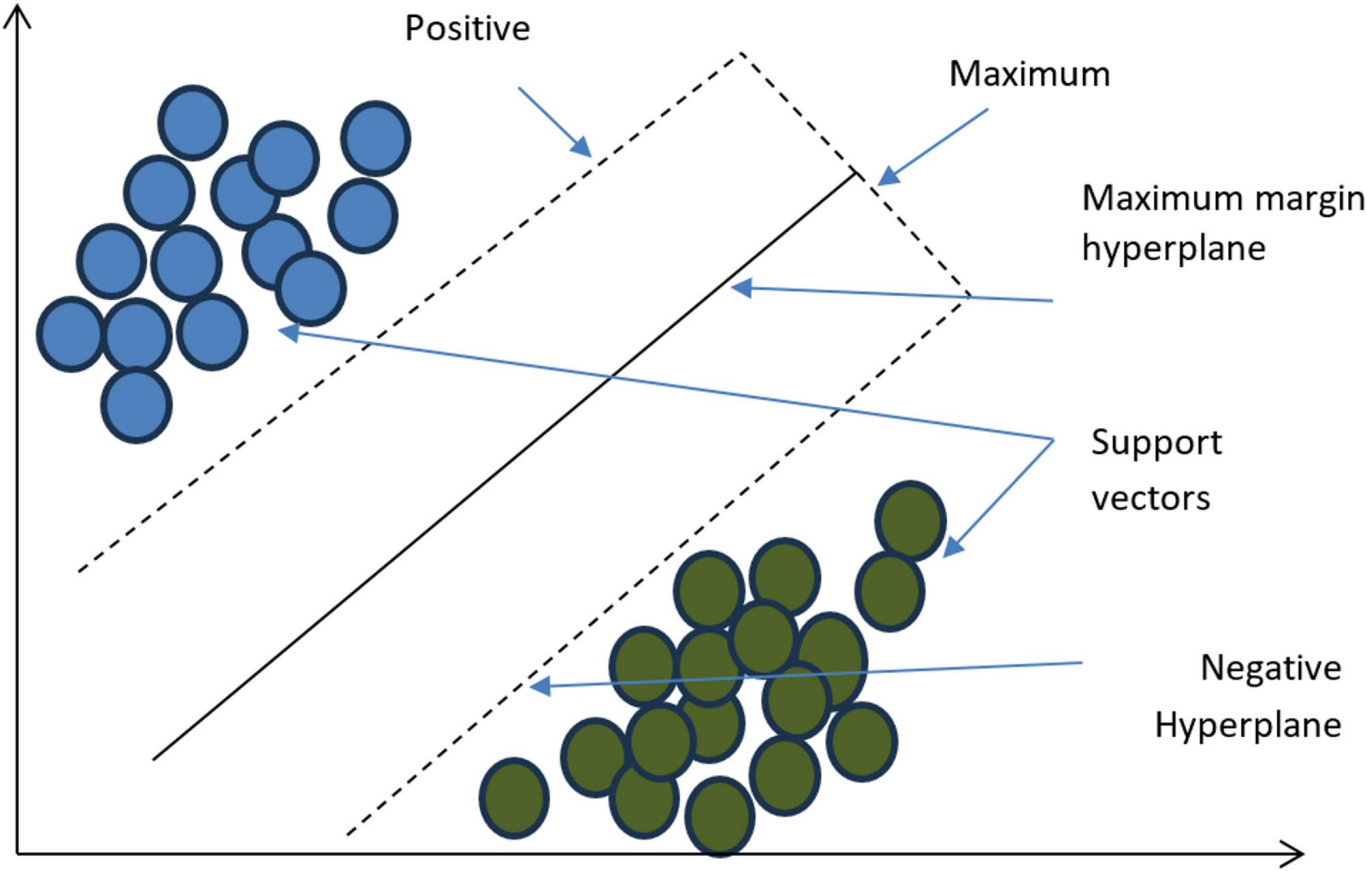
Key components of a Support Vector Machine (SVM).

A support vector machine (SVM) is used to supervise a learning algorithm that is better at classifying tasks by determining the optimal hyperplane, which separates data points that belong to other classes. It works on maximizing and increasing the margin and the distance between the hyperplane and the nearest data points, which increases the generalizability of unseen data, making it useful for high-dimensional spaces^22^. The SVM uses kernel functions with datasets that are nonlinearly separable, so the function transforms the input to a higher-dimensional space, so a linear separator is feasible. The kernel commonly used functions are the sigmoid, radial basis function (RBF), linear and polynomial functions. We choose which function works by knowing the problem and data characteristics. For example, the RBF is commonly used in medical diagnosis because it is flexible in modeling complex relationships, as noted in the literature^21^. We use the regularization parameter C in the algorithm because it controls the trade-off between maximizing the margin and minimizing classification errors. The flexibility of SVMs helps them work in many areas, such as bioinformatics, imaging recognition, and text classification^24^. Although the complexity of SVM training increases with large datasets, optimization or parallelized implementations can be used to handle this point. Overall, the SVM excelled in situations where the data had clear class boundaries or complex structures, as shown in Fig. 2.

### Artificial neural networks (ANNs)

**Fig. 3:**
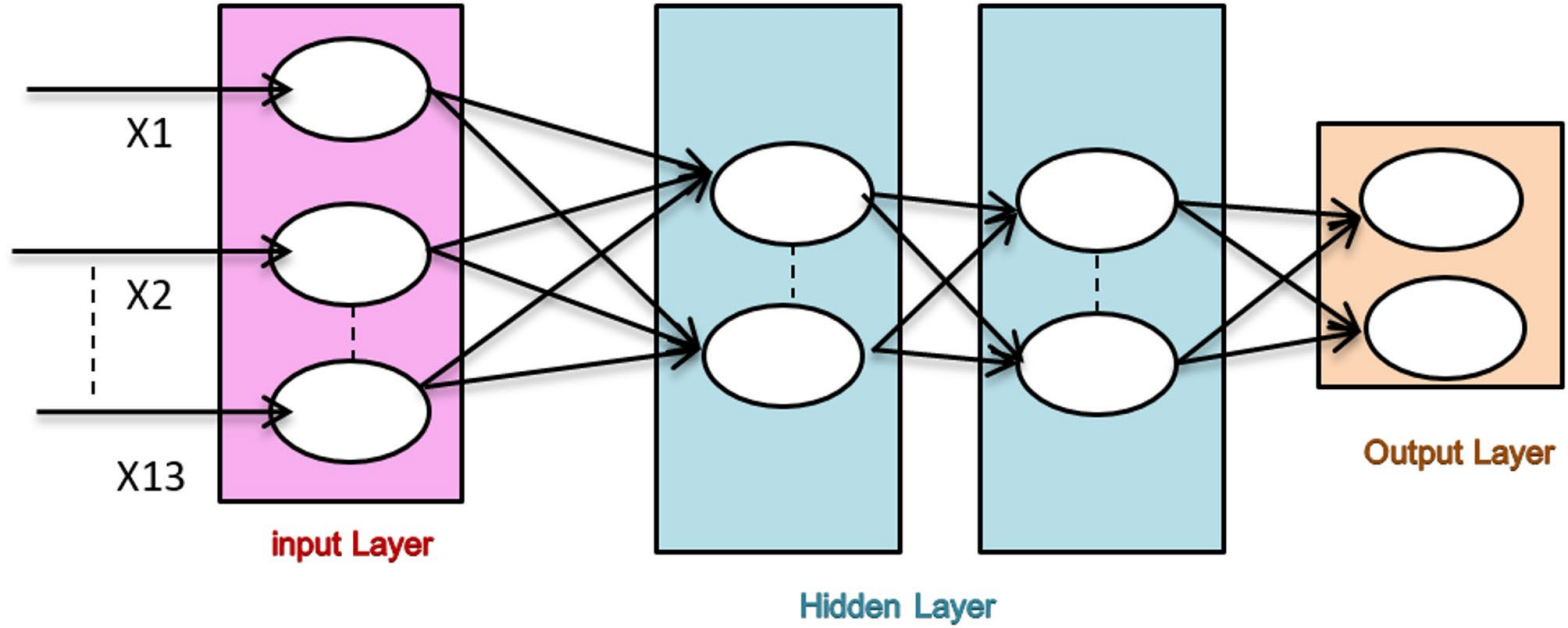
Structure of an Artificial Neural Network (ANN).

Because of their capacity to represent intricate nonlinear correlations in clinical information, artificial neural networks, or ANNs, have been widely used in the prediction of cardiac disease. The design of the ANN prediction model contains 13 neurons in the input layer, which represent clinical signs such as blood, pressure, cholesterol, age and sex. In addition, the ANN prediction model contains two hidden layers with ten and five neurons, which are used to capture complex patterns. The output layer with a single neuron is then used to determine whether cardiac disease is present, as shown in Fig. 3. These ANN models perform noticeably better in terms of prediction accuracy than conventional statistical methods do, according to recent studies^29,30^. By using past patient data, these models increase the accuracy of diagnoses and open the door to more individualized medical care, demonstrating how these ANN models perform noticeably better in terms of prediction accuracy than conventional statistical methods do. The accuracy of the model increases with respect to diagnoses when past patient data are used, so it helps in medical treatments.

### Long short-term memory (LSTM)

**Fig. 4:**
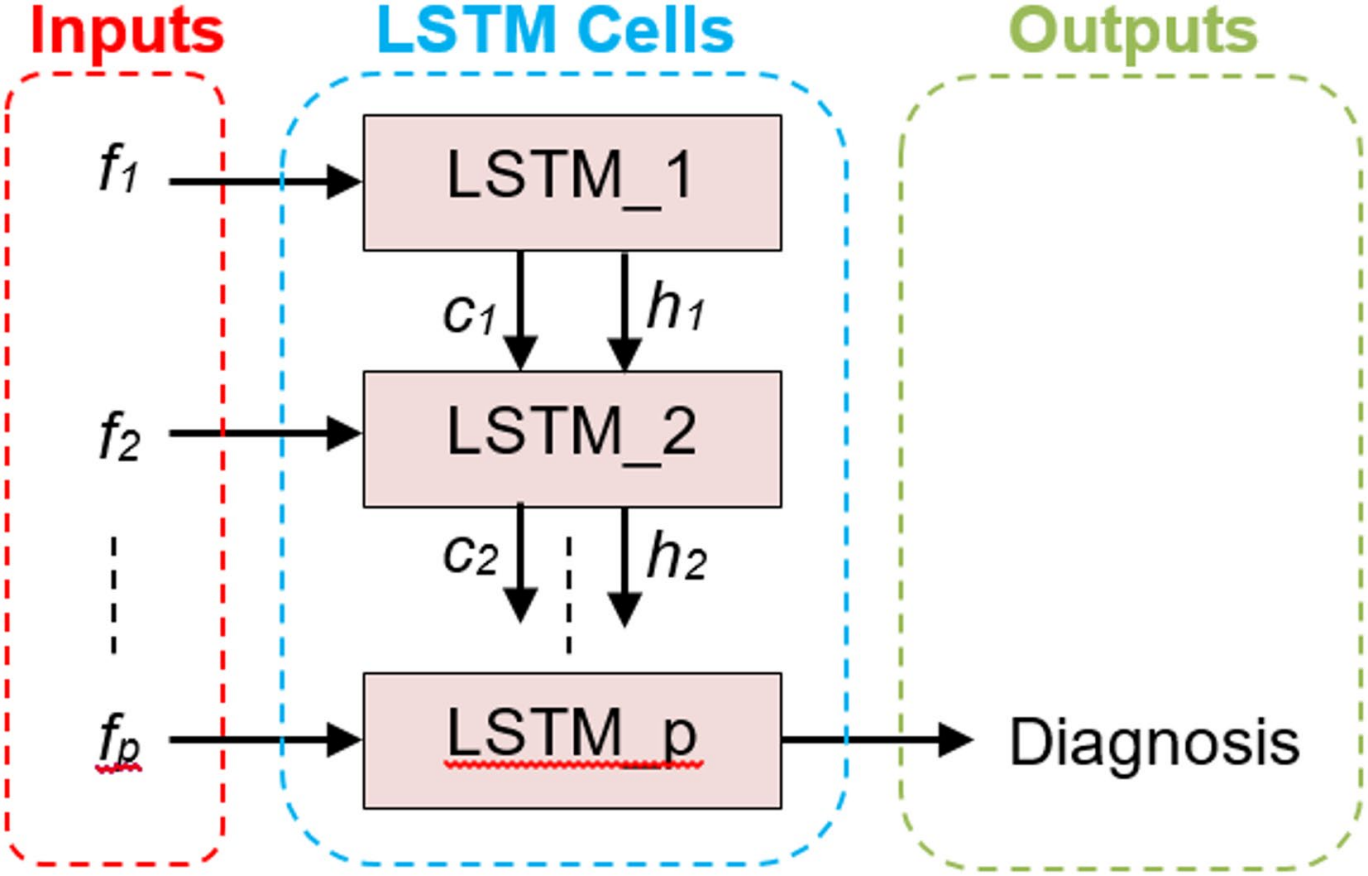
A many-to-one LSTM structure for multilabel diagnostics^31,32^.

In this section, we discuss the long short-term memory (LSTM) model, which is a deep learning framework method that is used to diagnose heart attack patients. By changing the memory cell with gates for hidden vectors in RNNs, LSTM is an advancement of RNNs that addresses the exploding issue and gradient vanishing^31,32^. Therefore, LSTM is a unique type of RNN that can recognize long-term dependencies. It also has the ability to retain information for extended periods of time by default. As a result of its capacity to learn from sequential data, LSTM is a widely used deep learning technology^31,32^. For a number of real-time applications, including medical diagnosis, phrase categorization, language modeling, sequence-to-sequence prediction, and different tagging methods, LSTM is an adequate model. As illustrated in Fig. 4, the model developed in this paper is built on a many-to-one LSTM structure to handle multilabel diagnostics.

**Fig. 5:**
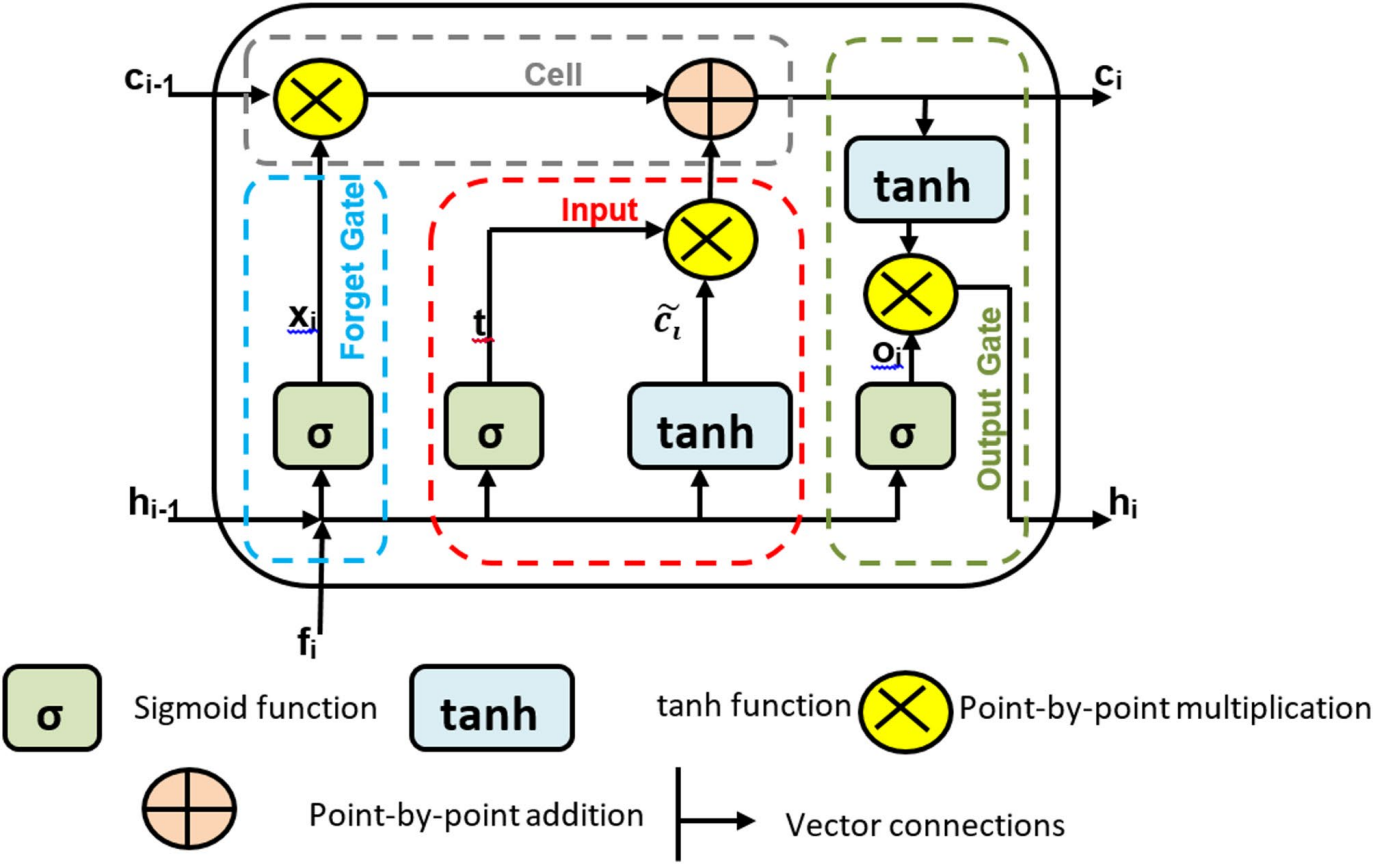
The structure of LSTM cell.

Figure 4 shows how the input dataset containing values of *‘p’* features is transmitted to *‘p’* LSTM cells, where the current output state (*h*_*i*_) and cell state *(c*_*i*_*)* of the *i*^*th*^ LSTM are used as inputs for the *(i + 1)*^*th*^ or subsequent LSTM. Stated differently, each LSTM’s outputs are fed into the subsequent LSTM, which in turn provides the final diagnosis. As shown in Fig. 5, each LSTM cell is made up of three gates: input, forget, and output gates. These gates are used to update the output value and preserve the cell state. The purpose of these gates is to regulate the information flow between cell states. To decide how to manage the flow data, sigmoid activation is used for each of the three gates (σ). Although information can be added or withdrawn via each gate, it actually does not change in the cell state. The input gate determines which input values should be used to alter the cell state. The output gate determines the quantity of output, and the forget gate determines the irrelevant information that should be left out of the cell state^31,32^.

Three primary processes are needed to build the LSTM. LSTM first starts to recognize unwanted data, which they forget gates, and then eliminates from the cell. The forget gate produces an output *(x*_*i*_*)* between zero and one by using the current input (*f*_*i*_) and the previous output *(h*_*i−1*_*)* in the cell state *(c*_*i−1*_*)*. One denotes utterly forgetting the information, whereas zero denotes fully remembering it. In the second stage, the input gate provides a judgment regarding the storage of information in the current cell state *(c*_*i*_*)* by multiplying its output *(t*_*i*_*)* by the output of the tanh activation layer ($\:\stackrel{\sim}{{c}_{i}}$). The flow of the proportion of information (*h*_*i*_) in the present cell state *(c*_*i*_*)* is given at the end of the third and last phases. by merging its output *(o*_*i*_*)* with the output of an additional tanh activation layer at the LSTM cell’s output via the output gate. These three gates’ functions in an LSTM cell for.

The mathematical representation of delivering output (*h*_*i*_) in cell state *(c*_*i*_*)* is (38–43)^31,32^.

38 $$\:{x}_{i}={\upsigma\:}({(w}_{x}*{h}_{i-1})+{(w}_{x}*{f}_{i})+{b}_{x})$$

39 $$\:{t}_{i}={\upsigma\:}\left(\right({w}_{t}*{h}_{i-1})+({{w}_{t}*f}_{i})+{b}_{t})$$

40 $$\:\stackrel{\sim}{{c}_{i}}=\text{tanh}({(w}_{c}*{h}_{i-1})+({w}_{c}*{f}_{i})+{b}_{c})\:$$

41 $$\:{c}_{i}={(x}_{i}*{c}_{i-1})+\:{(t}_{i}*\stackrel{\sim}{{c}_{i}})$$

42 $$\:{o}_{i}={\upsigma\:}\left(\right({w}_{o}*{h}_{i-1})+({w}_{o}*{f}_{i})+{b}_{o})$$

43 $$\:{h}_{i}={o}_{i}*tanh{\:c}_{i}$$

where the weight matrices are denoted by *w*_*x*_, *w*_*t*_, *w*_*c*_, and *w*_*o*_. The bias factors for the various LSTM gates are as follows. *b*_*x*_, *b*_*t*_, *b*_*c*_, and *b*_*o.*_

## Simulation and results

In this segment, we consider the EHAD strategy, in which an) is presented as a novel hybrid diagnostic strategy in the diagnosis of a heart attack. ECT combines three major records: (SVM)^18^, (LSTM)^19^, and (ANN)^20^. The combination of these models is conducted with the MV technique to arrive at a final decision concerning the usual heart disease dataset, which can be referred to as a commonly known dataset^33^, as depicted in Fig. 1. SVM is a classification model of the boundary type, LSTM is a method of learning on the basis of temporal sequences, and ANN is a general-purpose feedforward neural network. The three classifiers are first trained in parallel by using the same training subset and subsequently tested and proven by using the appropriate testing subset.

Finally, the MV technique will pick the diagnosis that will have the greatest number of agreements between the three classifiers. The implementation hypothesis is based on four steps that need to be carried out one by one, as listed in Fig. 1, and includes training, testing, validation, and final prediction by voting. The dataset was split with the help of a stratified 10-fold cross-validation mechanism. Additionally, 70/30 proportion was applied, with 70% of the data designated to be used in training and 30% to be used in testing. Standard scaling was performed for feature normalization via the StandardScaler function. No data balancing method (such as SMOTE) was used because the distribution of the data was already balanced. Region SVC with the probability setting turned on was used to implement the SVM. The ANN was built on the MLP classifier, the hyperparameters were tuned via GridSearchCV and 3-fold cross-validation, and early stopping was used with different options out of the whole size of the hidden layers, modes of solving, and learning rates.

The implementation of LSTM in Keras as a framework was performed with a reshaped 3D 2D input layer, a single layer of LSTM with 32 units, and a dense final layer with the activation pinned on the sigmoid. To train the model, 10 epochs with a batch size of 32 and binary cross-entropy loss were used. The MV approach was used after the prediction and was achieved by combining the outputs of the three models and taking the class to be agreed upon by at least two of them. The analysis of a strategy with standard performance measures reveals that the accuracy, recall, and precision are calculated on the basis of the confusion matrix given in Table 3. The formulas of these evaluation metrics are summarized in Table 4. The classifiers, both being tested separately and as an ensemble, were tested using the same testing set. Table 5 lists all the parameters and hyperparameters that were used to make each model reproducible and transparent in implementation. The ensemble-like EHAD strategy yields better results than the individual models do; therefore, the model diversity and use of voting rules are feasible for increasing the accuracy of heart disease diagnosis.

**Table 3:** Confusion matrix which depicts how diagnostic on cases.

	Diagnosed Label	
	Negative	Positive
Known Label		
Positive	False Negative (FN)	True Positive (TP)
Negative	True Negative (TN)	False Positive (FP)

**Table 4:** Confusion matrix formulas.

Measure	Formula	Meaning
Precision	TP/(TP + FP)	The percentage of positive diagnostics those are already correct.
Recall	TP/(TP + FN)	The percentage of positive diagnostics that were diagnosed as positive.
Accuracy	(TP + TN)/(TP + TN + FP + FN)	The percentage of diagnostics those are correct.
F1-measure	2*PR/(P+R)	The weighted harmonic mean of Precision and Recall.

**Table 5:** The used parameters and their values.

Technique	Parameter	Value/options
Svm	Probability	True (Enables probability estimates)
	Random_state	42 (Ensures reproducibility)
	Cross validation in Grid search	CV (k=3)
ANN	Hidden_layer_size	(32, 16), (64, 32) (Two architectures tested)
	Max_iter	1000,2000 (Max iterations for training)
	Learning_rate_init	0.001, 0.01 (Learning rates tested)
	Solver	‘adam’, ‘1bfgs’ (optimizers tested)
	Random_state	42 (For reproducibility)
	Early_stopping	True (Stops training if no improvement)
LSTM	Input shape	(1, number_of_features) (Reshaped inputs)
	LSTM units	32(Number of neurons in the LSTM layer)
	Learning_rate_init	(Adam default) 0.001
	Activation (LSTM)	‘relu’ (Activation function)
	Return_sequences	False (LSTM does not return sequences)
	Dense units	1 (Final output layer)
	Activation (Dense)	‘sigmoid’ (For binary classification)
	Optimizer	‘adam’ (Adaptive learning rate optimizer)
	Loss	‘binary _crossentropy’ (Loss function)
	Metrics	[‘accuracy’] (Tracks accuracy)
	Epochs	10 (Number of training rpochs)
	batch_size	32 (Mini-batch size)
Majority voting (Ensemble) Method)	Formula used	round((SVM_preds + ann_preds + lstm_preds)/3)(Averages predictions and rounds

Grid search in the space of predetermined hyperparameters is the main method used to perform hyperparameter tuning of artificial neural networks (ANNs). The combinations of the hidden_layer_sizes [(32, 16), (64, 32)], solver [‘adam’, ‘lbfgs’], max_iter [1000, 2000], and learning_rate_init [0.001, 0.01] were investigated during the search. Threefold cross-validation was applied as a method of performance evaluation, and the best configuration was chosen considering the validation accuracy and F1 score. The optimal values were as follows: hidden_layer_sizes = (64, 32), solver=’adam’, learning_rate_init = 0.001 and max_iter = 2000. The learning rate was chosen via this process of grid search, and early stopping was used to avoid overfitting, which provides a sort of implicit regularization. Although there is no dropout in practice (as the MLP classifier of scikit-learn is not capable of dropout), we are following up on this by adding dropout as an explicit regularization to deep learning approaches, such as Keras. In the case of the support vector machine (SVM), default hyperparameters were chosen (the kernel was the RBF), and in the future, it can be optimized to C, gamma, and kernel parameters via a randomized search technique or the grid pattern method. The LSTM model was developed in Keras with only one LSTM neuron and 32 units and a dense output layer with sigmoid activation. It was constructed via the Adam optimizer, binary cross-entropy loss, and default learning rate to complete 10 epochs with a batch size of 32. In this version, no deliberate hyperparameter optimization was applied on behalf of the LSTM, but the model architecture and approach parameters were selected on the basis of precedent empirical findings.

### Dataset 1 preprocessing

The statistical data utilized in the research included 303 records without missing values^33^. This was validated by means of an in-depth check of the data, which revealed that all of its attributes were filled in and ready to process. Continuous variables such as age, heart rate, CK-MB and troponin levels were normalized with min–max scaling to normalize the values between 0 and 1, which increases the performance of machine learning algorithms, especially ANN and LSTM. With respect to the target variable, a moderate class imbalance was detected: 61.2% of the records were positive (presence of heart attack), whereas 38.8% of the records were negative (absence of heart attack). To compensate for this, we used stratified 10-fold cross-validation so that the same class distribution was maintained in each fold. Moreover, precision, recall and F1-score metrics, which are more suitable for uneven datasets than for accuracy only, were used to assess performance.

### Dataset description

In this section, two datasets are described in detail.

#### Description of dataset 1

This dataset is commonly known as the “heart disease” dataset^33^, which has been widely used in cardiovascular research and machine learning studies. It comprises 303 patient records, each with 14 attributes related to heart health. The primary goal of this dataset is to predict the presence of heart disease in patients on the basis of these attributes. (1) Age: Age of the patient in years, (2) Sex: Gender of the patient (1 = male; 0 = female), (3) Chest Pain Type (cp.): 0: Typical Angina 1: Atypical Angina 2: Non-Anginal Pain and 3: Asymptomatic; (4) Resting blood pressure (trestbps): Resting blood pressure in mm Hg upon hospital admission; (5) Serum cholesterol (chol): Serum cholesterol level in mg/dl; (6) Fasting blood sugar (fbs): Fasting blood sugar > 120 mg/dl (1 = true; 0 = false), (7) Resting Electrocardiographic Results (Results): 0: Normal; 1: ST-T wave abnormality (e.g., T wave inversions and/or ST elevation or depression > 0.05 mV); and 2: probable or definite left ventricular hypertrophy according to Estes’ criteria, (8) Maximum heart rate achieved (thalach): maximum heart rate achieved during exercise; (9) Exercise-induced angina (exang): Exercise-induced angina (1 = yes; 0 = no), (10) ST Depression (old peak): ST depression induced by exercise relative to rest; 11. Slope of the Peak Exercise ST segment (slope): 0: Upsloping, 1: Flat and 2: Downsloping, 12. Number of major vessels colored by fluoroscopy (ca.): Number of major vessels (0–3) colored by fluoroscopy, 13. Thalassemia (thal): 1: normal, 2: fixed defect and 3: reversible defect, 14. Target: Diagnosis of heart disease (0 = absence; 1 = presence).

**Fig. 6:**
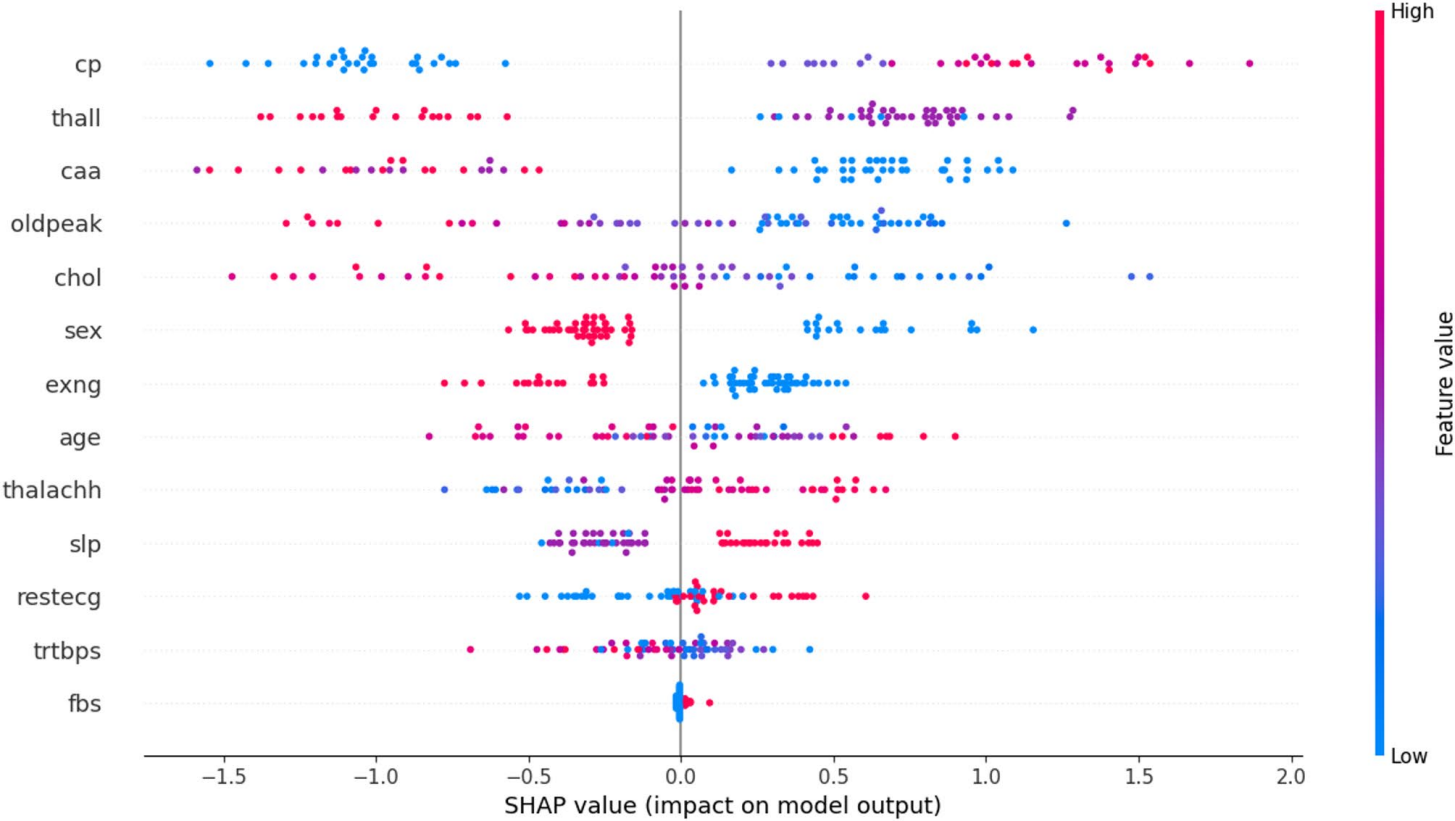
SHAP dot plot of feature contributions.

To better understand the contribution of the dataset features to the prediction process, we applied SHAP (SHapley Additive exPlanations) analysis using an XGBoost model trained on the dataset. The SHAP feature importance results Fig. 6 indicate that variables such as age, cholesterol, resting blood pressure, and maximum heart rate had the highest impact on prediction outcomes. Other features, including fasting blood sugar, ST depression (oldpeak), and exercise-induced angina, also contributed meaningfully but to a lesser extent. Features such as sex and resting ECG showed relatively lower influence. This analysis provides an initial understanding of the dataset characteristics, highlighting which clinical attributes are most relevant to heart disease risk. Incorporating SHAP at this stage supports transparent reporting of feature importance and complements the subsequent modeling and evaluation process.

#### Description of dataset 2

The given dataset, known as the “heart attack dataset”^40^, covers elaborate clinical and diagnostic data of 1,319 individuals, concentrating on indicators that are highly relevant to the state of the cardiovascular organism, especially in the case of possible heart-related disorders. It contains nine factors both in the category of demographic variables and biomarkers, and it is designed to answer binary classification tasks on the basis of a target variable called Result and denotes whether the patient is diagnosed as positive or negative with a condition that is presumably a cardiac event, possibly a myocardial infarction. The demographic variables cover five categories, i.e., Age (14–103) and Gender (0 and 1, presumably female and male, respectively). The age distribution within the dataset is fairly even, with a mean age of approximately 56 years, representing the elderly to middle-aged population of patients, which is normally associated with a greater risk of cardiovascular repercussions. Heart rate, systolic blood pressure and diastolic blood pressure are used to indicate vital signs. These factors are critical in the evaluation of cardiovascular functionality.

The heart rate values are rather variable, ranging from 20 beats per minute to 1111 beats per minute, indicating the possibility of outliers or even false entries. Blood pressure measurements are also very broad: the values of systolic blood pressure are between 42 and 223 mmHg, and diastolic blood pressure is between 38 and 154 mmHg, which includes hypotensive and hypertensive disorders. Examples of biochemical markers are blood sugar, CK-MB and troponin, all of which play important roles in determining a disease such as acute coronary syndrome. Blood sugar changes between 35 and 541 mg/dl, with normal, prediabetic and diabetic levels. The level of creatine kinase-MB (CK-MB), a heart enzyme released during damage to the heart, ranges from 0.321 to 300, whereas the level of troponin, which is used as a more specific cardiac injury marker, ranges from 0.001 to 10.3. These broad scopes of data imply that the population consists of normal and pathological cases. There are two values in the target column, i.e., “positive” and “negative”, which indicate unequal data distributions; the strength of the positive category (or the majority of the data, 810 in 1319 cases) opportunity recommends the use of class balancing tasks during model training. The organized character of the dataset, the absence of missing values, is adequate to allow the use of these data in machine learning tasks, including predictive modeling, risk stratification, or a clinical decision support system in cardiology.

### Testing the EHAD strategy against other techniques on dataset 1

**Fig. 7:**
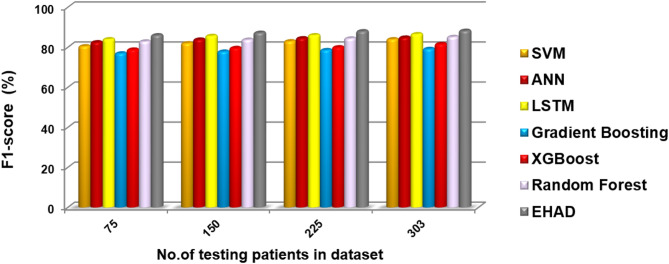
F1-score percentage results, showing EHAD’s stronger balance between precision and recall.

**Fig. 8:**
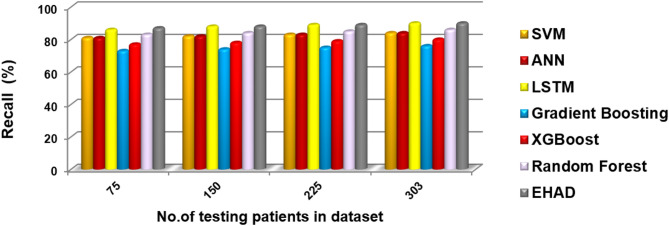
Recall percentage comparison, highlighting each model’s sensitivity.

**Fig. 9:**
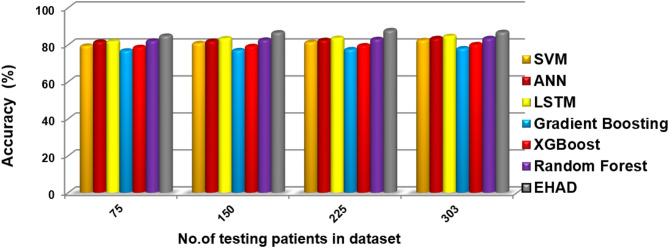
Accuracy percentage values, indicating overall prediction correctness.

**Fig. 10:**
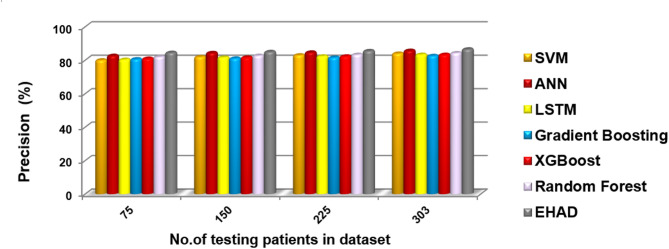
Precision percentage of the tested techniques reflecting reliability of positive predictions.

**Fig. 11:**
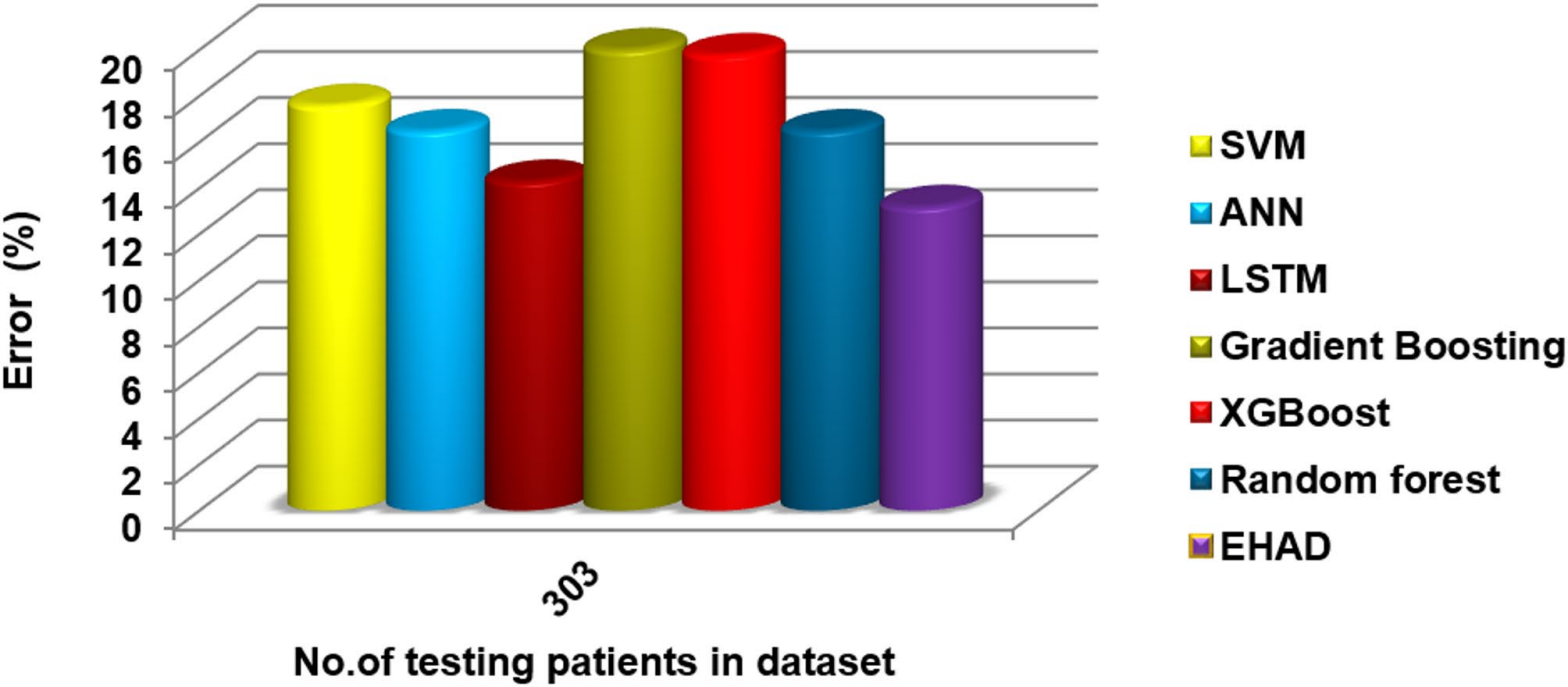
Error percentage results, reflecting the reliability of positive predictions.

In this segment, the EHAD strategy is executed and compared with other diagnostic techniques to ensure that EHAD can provide fast and accurate diagnoses. These techniques include SVM^22^, ANN^29,30^, LSTM^31,32^, gradient boosting^13^, XGBoost^11^, random forest^11^ and ensemble classification (ECT) techniques. In fact, the EHAD model is applied on the basis of valid data without useless features to obtain quick and correct results. The accuracy, error, precision, recall, and F1-measure calculations are illustrated in Table 5. The implementation time measurement is also provided in ig1***2. Finally, the classification according to the confusion matrix formulas for the testing dataset is illustrated in Figs. 7, 8, 9 and 10, and 11 to prove the computational efficiency of the proposed EHAD strategy over other strategies. EHAD provides the best performance values; thus, it outperforms the other techniques.

Figures 7, 8, 9, 10, and 11 present the estimated performance metrics—accuracy, precision, recall, and F1 score—for four models (SVM, ANN, LSTM, and majority voting) (EHAD) across varying dataset sizes: 75, 150, 225, and 303. For a dataset size of 75, the SVM achieves an accuracy of 79.4%, with a precision of 80% and a recall value of 81%, resulting in an F1 score of 80.5%. The ANN shows slightly better performance, with an accuracy of 81.6%, and the LSTM model excels in terms of recall at 81%, with an accuracy of 81.8%, leading to an F1 score of 82.5%.

The majority voting method, which combines the predictions of all the models, performs best among the groups at this size, with an accuracy of 86.81%. At the base dataset size of 150, the SVM achieves an accuracy of 80.8%, whereas the ANN improves to 82%, and the LSTM reaches an accuracy of 83.4%. The majority voting method has advantages over the other methods, with an accuracy of 86.6%. When the dataset size increases to 225, all the metrics increase across all the models, but the majority voting method achieves an accuracy of 87.8%, which reflects the benefit of the ensemble. Finally, when the dataset size was increased to 303, improvements in all the models were observed.

The accuracy was 82.42%, the error rate was 17.58%, the specificity was 80.49, the MCC was 64.49, and the ROC-AUC was 88.54. Moreover, to a lesser extent, the ANN model demonstrated superior performance, with an error rate of 16.48%, accuracy of 83.52%, specificity of 82.93%, MCC of 66.80%, and ROC-AUC of 88.63. The LSTM model achieved high-quality classification, especially in terms of sensitivity, with the best recall of 90%. It was also able to achieve an accuracy rate of 84.62%, specificity of 82.93%, and MCC of 71.10%, as well as an ROC-AUC of 89.39%, which meant that it had a good balance of detecting positive samples and falsely detecting negative samples. The majority voting ensemble (EHAD) was the best performing model, with the highest accuracy of 86.81%, specificity of 85.37, MCC of 75.50 and ROC-AUC of 89.51. This ensemble approach was successful in using the capabilities of separate classifiers (SVM, ANN and LSTM) and strengthened the robustness and generalization capability of the model. Table 6 provides a detailed overview of the performance indicators, in which not only accuracy and recall are used but also specificity, the MCC, and the ROC-AUC are used; it has also been confirmed that the majority voting strategy is optimal compared with individual models in the problem of heart disease diagnosis.

**Table 6:** Comparison between diagnosis models.

Metrics	Recall (%)	Precision(%)	F1-score (%)	Accuracy (%)	Specificity(%)	MCC(%)	ROC-AUC	Time (Sec.)
SVM	84	84	84	82.42	80.49	64.49	88.54	0.02
ANN	84	85.71	84.85	83.52	82.93	66.80	88.63	1.39
LSTM	90	83.33	86.54	84.62	82.93	71.1	89.39	3.29
Gradient Boosting	76	82.61	79.17	78.02	80.49	56.2	87.27	0.10
XGBoost	80	83.33	81.63	80.22	80.44	60.3	87.22	20.84
Random Forest	86	84.31	85.15	83.52	80.39	66.7	89.00	0.19
EHAD	90	86.54	88.24	86.81	85.37	75.5	89.51	4.7

**Fig. 12:**
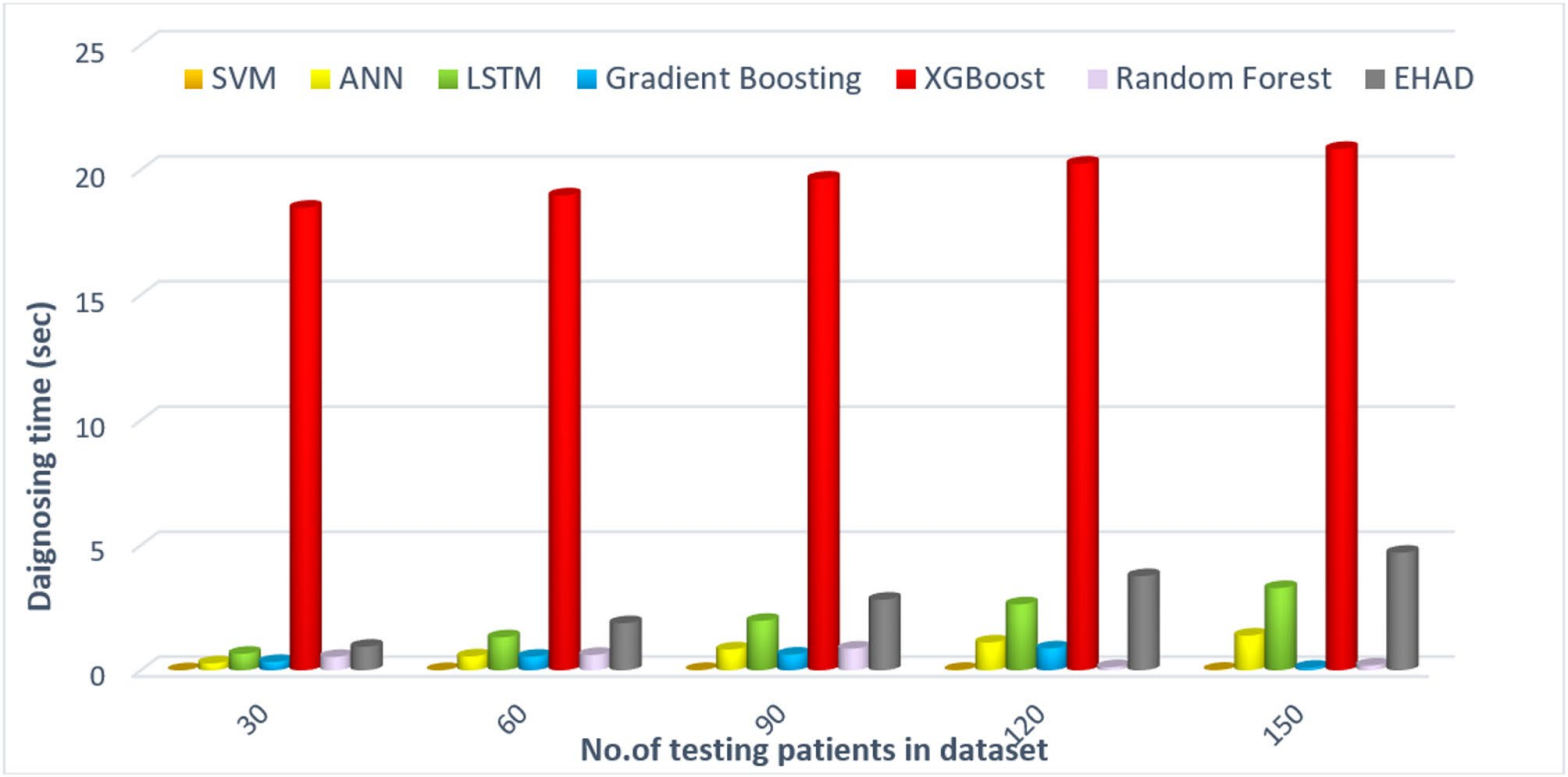
Average computation time (s), comparing model efficiency.

Figure 12 shows the estimated prediction times for the majority voting method across various dataset sizes: 30, 60, 90, 120, and 150 samples. For each dataset size, the prediction time for each individual model, called the SVM, ANN, and LSTM, is presented, followed by the total voting time. For example, with a dataset of 30 samples, the SVM takes approximately 0.004 s, the ANN takes approximately 0.278 s, and the LSTM takes approximately 0.659 s, resulting in a cumulative voting time of 0.941 s. As the dataset size increases, the prediction times for each model also increase incrementally. For 150 samples, the SVM prediction time reaches 0.02 s, the ANN prediction time is 1.39 s, the LSTM prediction time is 3.29 s, and the EHAD prediction time is 4.7 s. Overall, the table illustrates that while the majority of the voting process involves multiple models, the total time remains relatively quick compared with the individual training times, highlighting the efficiency of aggregating predictions in the ensemble methods. However, the execution time of EHAD is greater than that of other techniques, but it is the most accurate model where its execution time is neglected compared with its accuracy.

A variety of models based on boosting techniques, including gradient boosting^13^, XGBoost^11^, and random forest^11^, are tested and compared with the proposed EHAD, as shown in Table 6. The gradient boosting model has moderate performance and 78.02% accuracy, a recall of 76%, a specificity of 80.49%, an MCC of 56.2 and an ROC-AUC score of 87.27 but still has a small execution time of 0.10 s. Conversely, XGBoost led to improvements in accuracy and precision, with an accuracy rate of 80.22, recall of 80, specificity of 80.44, MCC of 60.3, and ROC-AUC of 87.22. However, it took the longest execution time, 20.84 s, which can affect the efficiency of the program in terms of real-time programs. The random forest model also achieved a balanced performance, with a recall of 86%, accuracy of 83.52%, specificity of 80.39%, and MCC of 66.7, with an ROC-AUC of 89.00, which took 0.19 s; these parameters illustrate that the RF model is effective and fairly quick. Finally, the EHAD ensemble method outperforms the individual models in all the measures. It had the best accuracy of 86.81%, recall of 90%, specificity of 85.37%, MCC of 75.5%, ROC-AUC of 89.51%, and acceptable execution time of 4.7 s. These findings indicate the usefulness of the majority voting ensemble in highly enhancing diagnostic performance, whereas it does not cause an impractical performance time in predicting heart disease, as shown in Table 6.

### Testing the EHAD strategy against other techniques on dataset 2

With dataset 2, which has 1319 records, the performance of the models is improved significantly. The SVM model achieves an accuracy of 78.79 against an error rate of 21.21%, whereas the ANN model also achieves an accuracy of 77.78 against an error of 22.22%. Notably, the ANN model has the highest recall of 84%, which means that it is powerful in detecting positive cases. The LSTM model has an accuracy rate of 79.55%, whereas the corresponding error rate is 20.45%. Gradiant boosting achieved a precision of 82.10%, an accuracy of 79.20%, and an error of 20.8%. XGBoost achieved higher accuracy and precision values of 79.50% and 82.70%, respectively. The random forest model achieves the highest accuracy for a single technique, with a value of 79.90% and an error of 20.1%. With respect to all the models, the majority voting technique (EHAD) still has the best output data, with the highest accuracy rate of 80.05 and the lowest error rate of 19.95. This indicates its usefulness in integrating several classifiers to increase its prediction performance. Overall, Table 7 shows a comparative assessment of the metrics of all the models, which confirms that the ensemble (EHAD) method yields consistent enhancements with increasing data size.

**Table 7:** Comparison between diagnosis models.

Metrics	Recall (%)	Precision(%)	F1-score (%)	Accuracy (%)	Specificity (%)	MCC (%)	ROC-AUC (%)	Time (Sec.)
SVM	82	83.26	82.57	78.79	76.26	55.5	86.24	4.5
ANN	84	80.39	82.33	77.78	75.32	52.6	87.12	1.69
LSTM	83	83.47	83.30	79.55	76.47	55.1	87.36	6.05
Gradient Boosting	82	82.10	82.05	79.20	76.60	55.5	87	0.29
XGBoost	83	82.70	82.84	79.50	76.70	55.7	87.26	19.61
Random Forest	83.5	83.1	83.30	79.90	76.74	56	87.34	0.34
EHAD	84	83.61	83.78	80.05	76.77	56	87.73	12.24

### Statistical analysis

To evaluate the diversity and agreement among the individual models of the ensemble, we calculate both the Q statistic and the Cohen kappa coefficient of 2 models of each pair of classifiers. The Q statistic between SVM and ANN was 0.813, and it was higher, but not so much, between SVM and LSTM, at 0.937, which shows that there was strong agreement but did not eliminate diversity. The Q statistic of 0.813 was also common to both the ANN and LSTM models. These values imply values of complementary behaviors between classifiers, not redundancy. Similarly, the values of Cohen’s kappa were 0.665 when the SVM and ANN were compared, 0.779 when the SVM and LSTM were compared, and 0.667 between the ANN and LSTM, which indicates high agreement between classifiers. This variance, especially as expressed through Q statistics, is essential to ensemble learning, as it forms the reasoning behind the adoption of majority voting in that it can be established that the classifiers help provide a unique point of view in the ensuing decision.

For dataset 1, we used stratified 10fold cross-validation to encourage statistical robustness and reduce overfitting in all the models of dataset 1. This method maintains the level of each fold in the classes, which is a key factor in medical data, where imbalance occurs between classes. The metrics used to evaluate it are the accuracy, precision, recall, F1 score, Matthews correlation coefficient (MCC), and ROC-AUC. The majority voting ensemble was superior to the individual models on some of the most important metrics. It has the maximum accuracy of (82.50% +/− 5.56), followed by the SVM (81.51% +/− 5.38), ANN (81.17%+/− 6.2), and LSTM (7.88-/+80.87). In terms of the F1 score, the ensemble was the best once again at 85.03% +/− 4.59, followed closely by the LSTM with 84.04% +/− 5.12, the ANN with 84.08% +/− 4.2 and the SVM with 6.8-/+83.44. In the case of recall, the highest value was 90.91% (+/− 4.79 and +/− 6.43 standard deviations, respectively) for the ensemble and LSTM, compared with 89.09% +/− 5.50% for the ANN and 88.48% +/− 7.23% for the SVM. In terms of precision, the ensemble method had the highest precision (80.05% +/− 7.26), followed closely by the LSTM and ANN methods (79.98% +/− 5.61) and (78.98% +/− 6.83), respectively, with the lowest score to the SVM method (78.32% +/− 5.68%). The model ensemble method also had the greatest MCC (0.651 +/− 0.1128) compared with the LSTM (0.6326 +/− 0.1076), ANN (0.6264 ± 0.1277) and SVM methods. (0.6156 +/− 0.1615)

Similarly, the ensemble model had the best ROC-AUC score (0.8165+/− 0.0579) compared with the LSTM (0.8072 +/− 0.0581), ANN (0.8018 +/− 0.0644), and SVM (0.8012 +/− 0.0795) models.

For dataset 2, to be statistically robust and reduce the possibility of overfitting, in this research, we implemented stratified 10-fold cross-validation on all the models. The approach preserves the global class balance among folds, which is especially important when dealing with medical data that lack class balance. The support vector machine (SVM) obtained a mean accuracy of 76.4% +/− 4.3%, a recall of 79.3% +/− 4.6%, a precision of 80.1% +/− 4.1%, and an F1 score of 79.5% +/− 4.0%. The ANN obtained an accuracy of 75.7% +/− 4.6, a recall of 80.3% +/− 4.4, 78.8% +/− 4.3 precision and an F1 score of 79.3% +/− 4.2. The LSTM model thus fared higher in terms of the F1 score. 80.6% +/− 3.6, with an accuracy of 77.1% +/− 4.0, a recall of 80.9% +/− 4.1 and a precision of 80.4% +/− 3.8. Gradient boosting and XGBoost achieved very good results, with accuracies of 77.3% +/− 3.6% and 77.5% +/− 3.4%, respectively, and F1 scores of approximately 80%, with high sensitivity and specificity. The random forest model provided even more moderate results, with an accuracy of 77.80% +/− 3.1 and an F1 score of 80.60% +/− 3.0. Notably, the EHAD ensemble model was the most effective since it performed better than all the single models in terms of nearly all the evaluation measures, with an accuracy of 82.50% +/− 3.2, a recall of 85.30 +/− 3.5, a precision of 84.90 +/− 3.1 and an F1 score of 85.60 +/− 3.4 being the highest. These findings affirm the efficacy of the ensemble method of EHAD in pooling the predictive abilities of the base classifiers and increase the generalizability and reliability of the heart disease diagnosis framework.

**Fig. 13:**
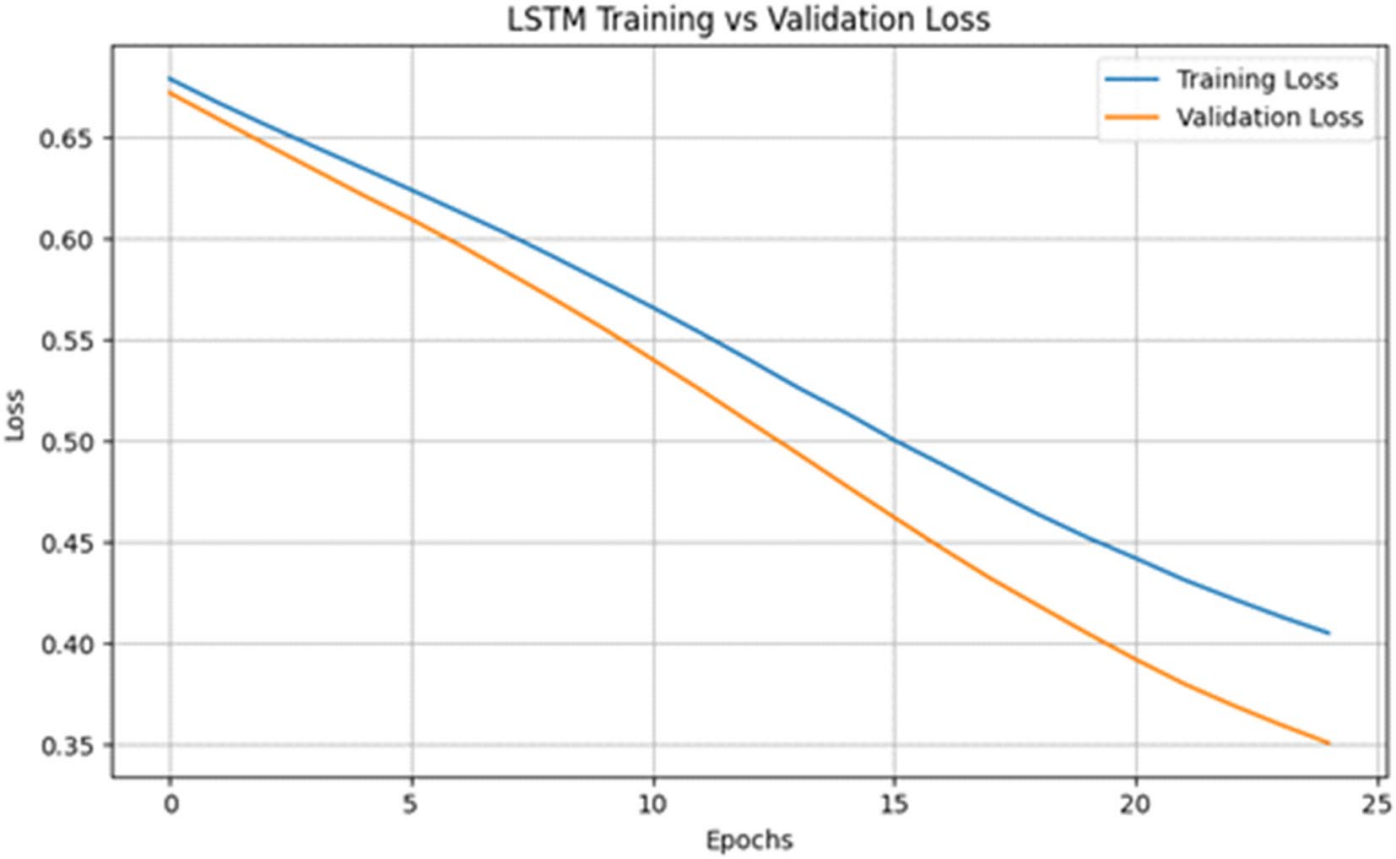
LSTM training vs. validation loss.

Figure 13 shows the training and validation loss curves of the LSTM model, as shown in Fig. 13, over 25 epochs. The losses steadily decrease, and the validation loss closely follows the training loss, which means that we have a well-generalized model and that overfitting is minimal. The trend of the line that indicates a decrease in loss in time indicates that the model was able to learn inherent patterns of the input features and still managed to maintain generalization ability on unseen data. This convergence pattern also suggests that selected hyperparameters, such as the learning rate and batch size, are suitable for stabilizing training. This type of behavior is characteristic of successful learning, in which the model performance on the validation data increases even more rapidly than the training performance does, as expected because of the effectiveness of the regularization techniques and early stopping criterion^34^.

**Fig. 14:**
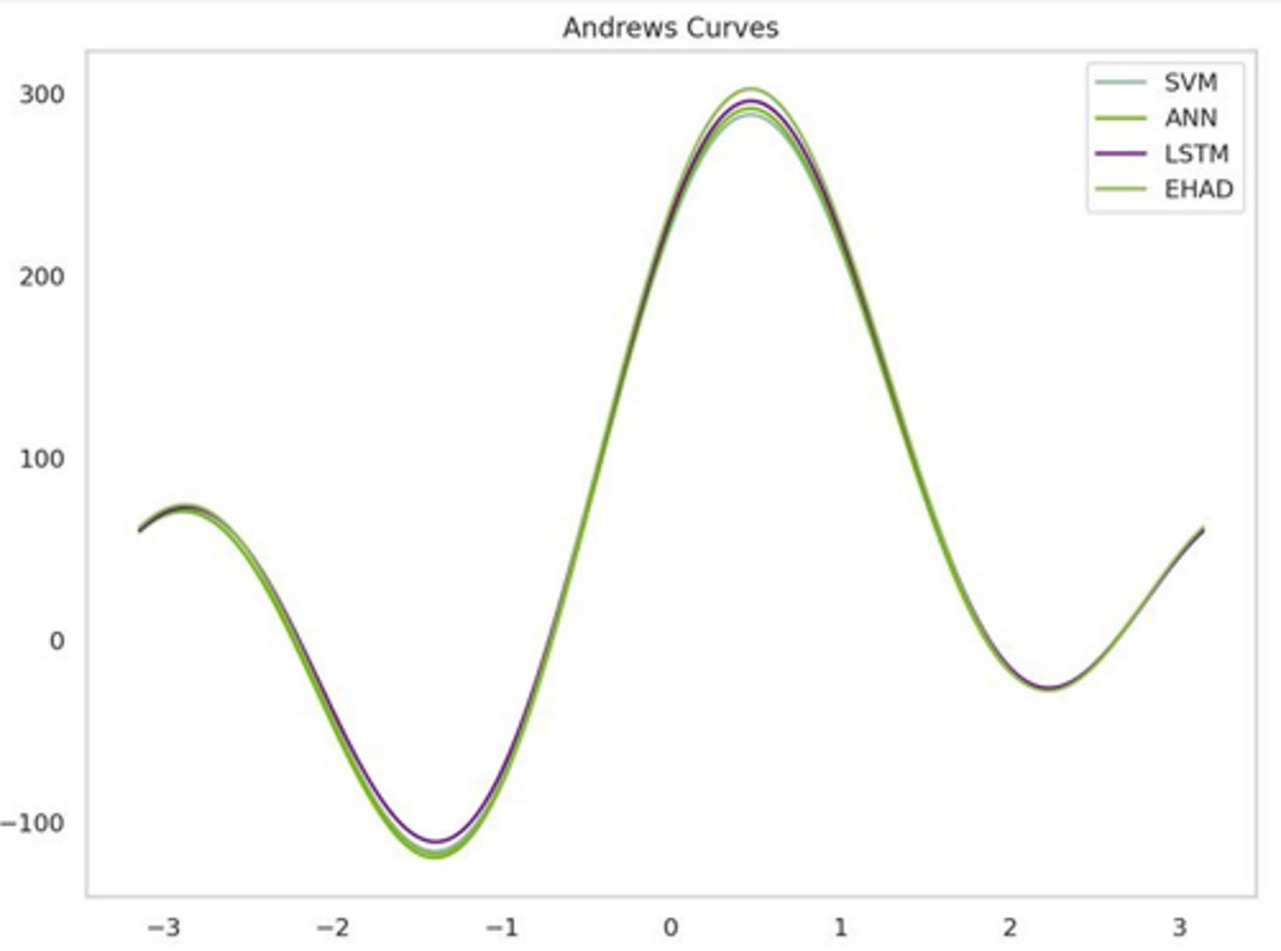
Andrew curves.

Second, Andrews curve visualization uses a trigonometric function to convert high-dimensional data into a two-dimensional curve^35^. A model (SVM, ANN, LSTM, or EHAD) over several metrics is represented by each line. Similar wave patterns are displayed by all the models, suggesting similar performance trends. Nonetheless, EHAD exhibits a marginally greater amplitude, particularly in the vicinity of the peak, indicating that it consistently performs better in terms of specific criteria. The robustness of EHAD across several performance metrics is vividly highlighted by this curve, as shown in Fig. 14.

**Fig. 15:**
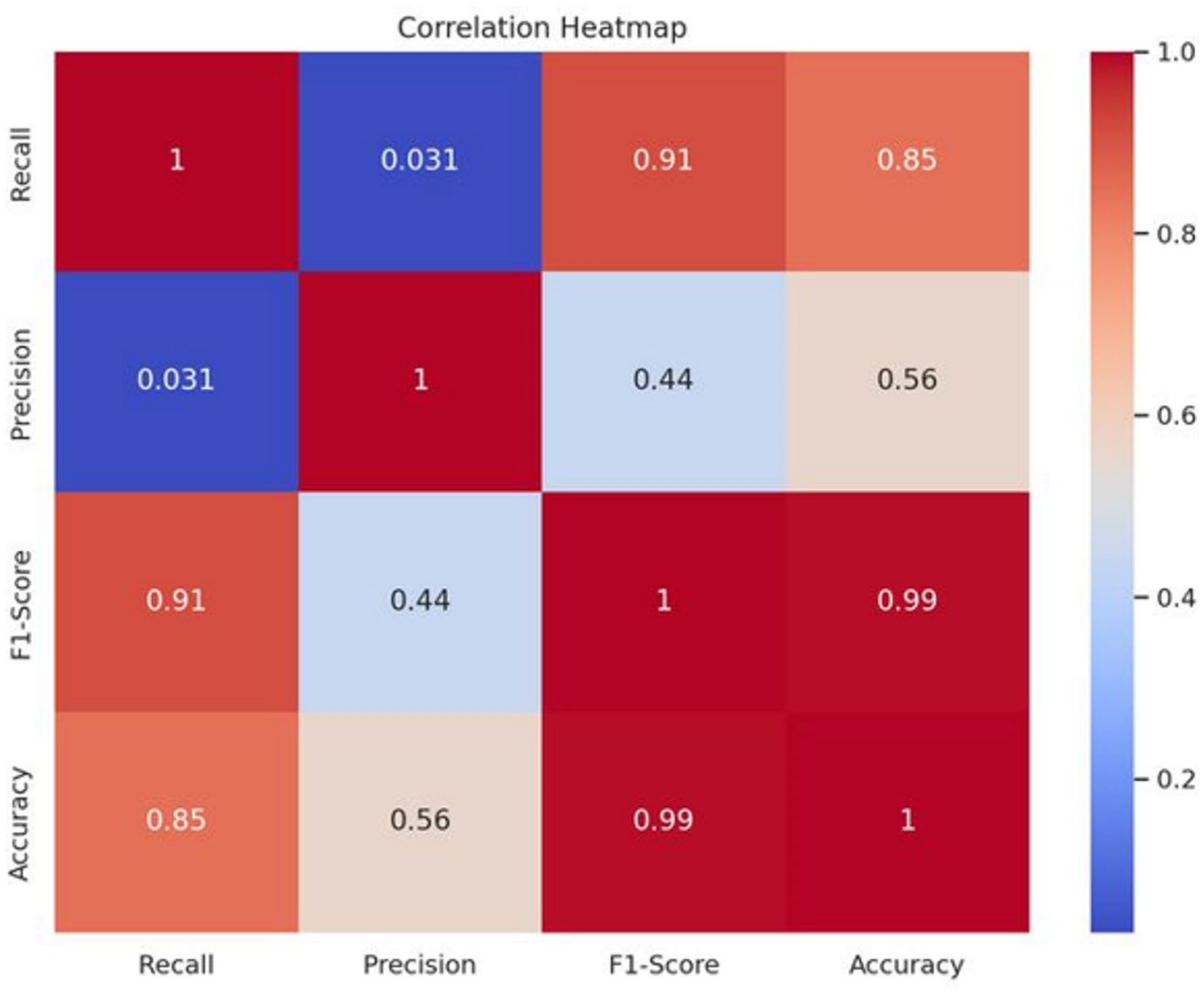
Correlation heatmap.

The third correlation heatmap^36^ displays the Pearson correlation coefficients between four performance metrics: accuracy, precision, F1 score, and recall. The color intensity of the correlations indicates how strong the relationship is; accuracy and F1 score have a nearly perfect correlation (0.99), meaning that improving one is likely to improve the other; recall and precision have almost no correlation (0.031), meaning that models that are strong in one may not necessarily perform well in the other. This differentiation aids in identifying trade-offs, as shown in Fig. 15.

**Fig. 16:**
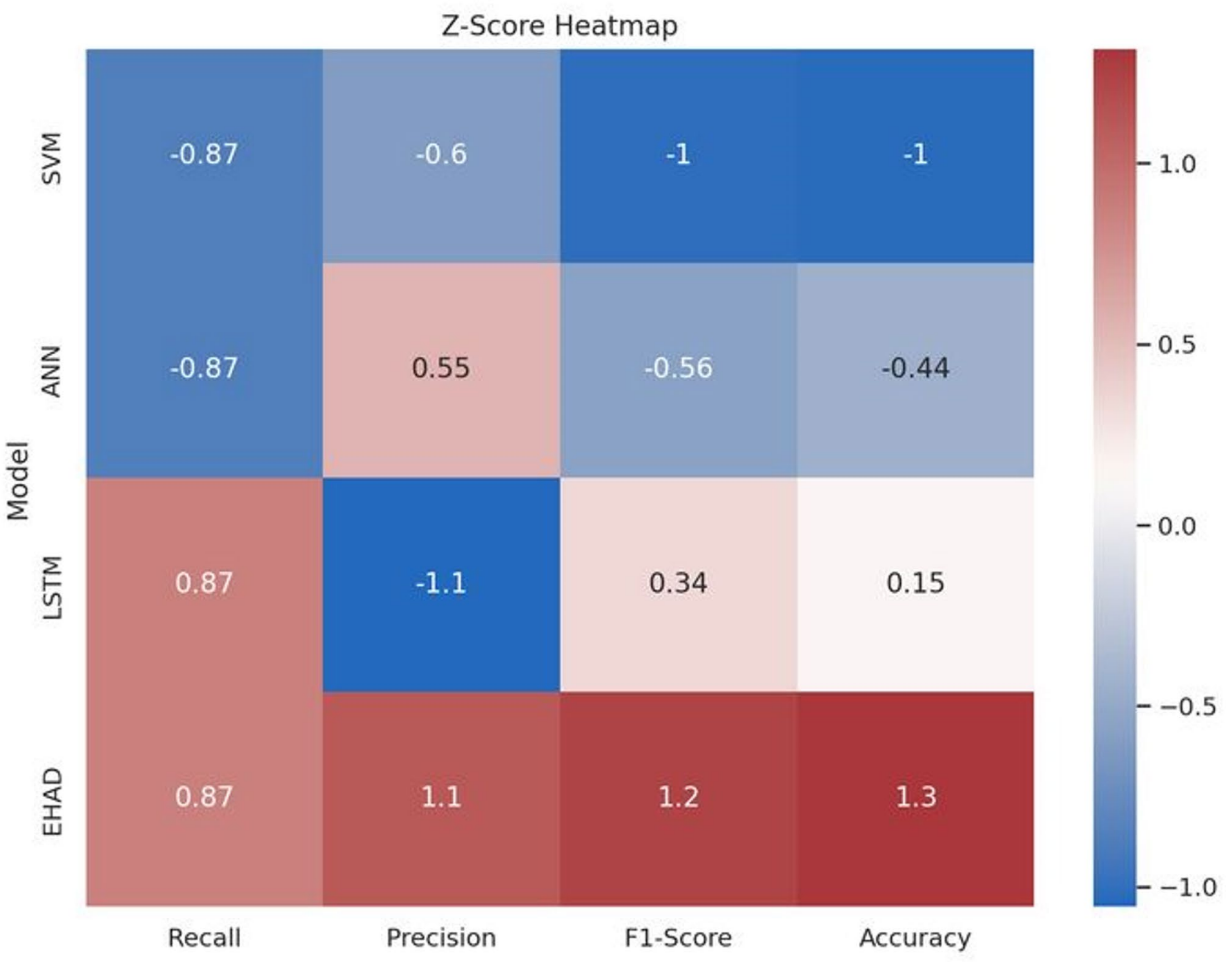
Z score heatmap.

The fourth heatmap of the Z score^37^ for each model across the four metrics is shown as the point’s distance from the mean. With continuously high positive Z scores (particularly in precision, F1 score, and accuracy), EHAD performs better than the other methods do, whereas SVM has the lowest ranking with all negative values. The performance of the ANN and LSTM is modest. Z scores emphasize EHAD’s outstanding position and enable normalized comparisons, as shown in Fig. 16.

**Fig. 17:**
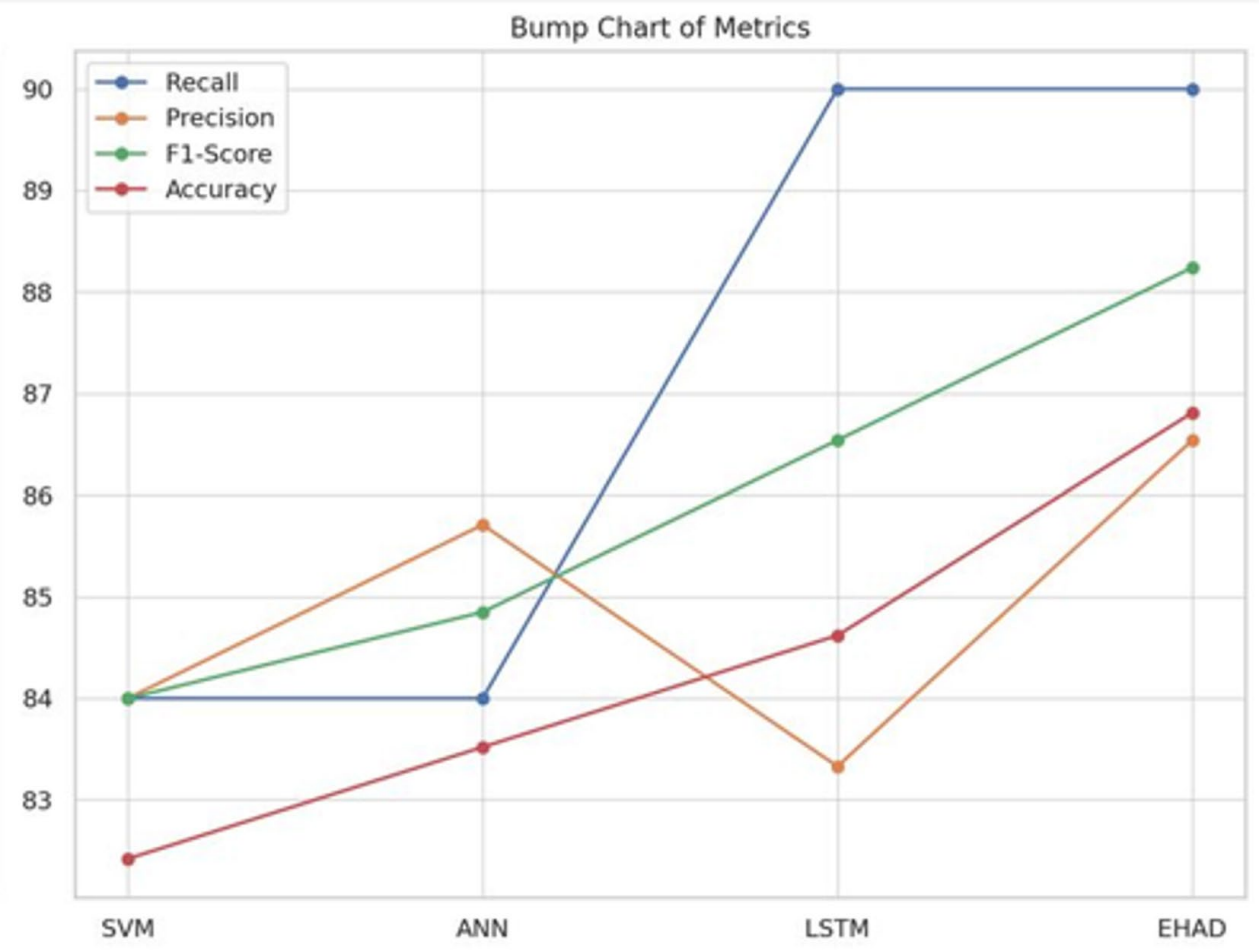
Bump chart of metrics.

Fifth Metrics Bump Chart^38^. Each model’s rank position across several performance measures is displayed visually in a bump chart. The models are listed on the X-axis, whereas the metric values are displayed on the Y-axis. In terms of most measures, particularly precision and accuracy, EHAD is at the top, followed by LSTM. SVM consistently has the lowest ranking and barely varies among the metrics. This graphic does a good job of conveying the models’ relative strengths and performance changes, as shown in Fig. 17.

**Fig. 18:**
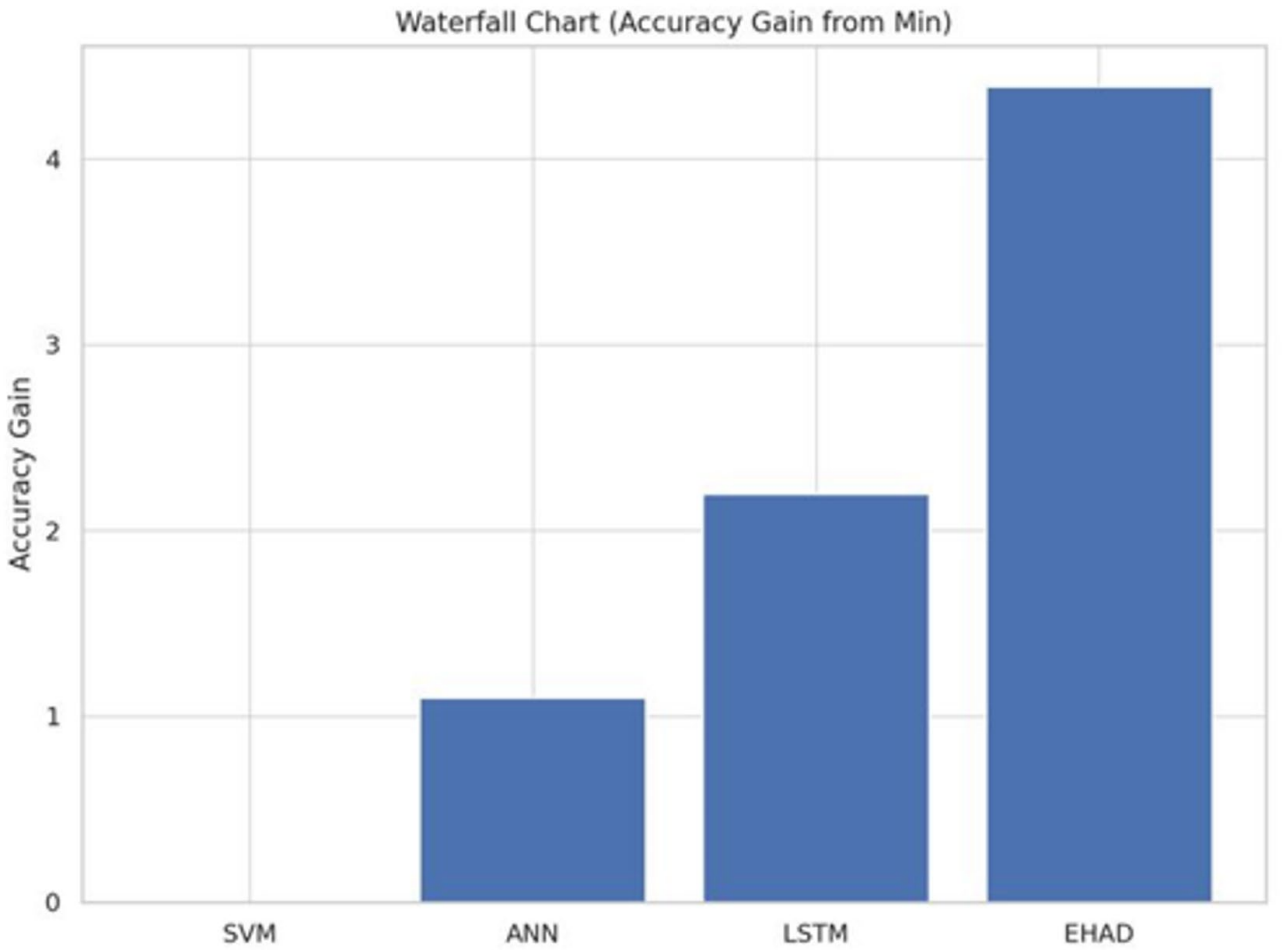
Waterfall chart.

Sixth accuracy gain from the Min Waterfall Chart^39^. Each model’s incremental accuracy gains in comparison to the baseline (SVM), the model with the lowest performance, are displayed in the waterfall chart shown in Fig. 18. With an accuracy improvement of 4.39%, EHAD outperforms ANN and LSTM. These step-by-step graphics highlight the efficiency of EHAD in increasing classification accuracy and reveal how much better each model performs than the baseline.

**Fig. 19:**
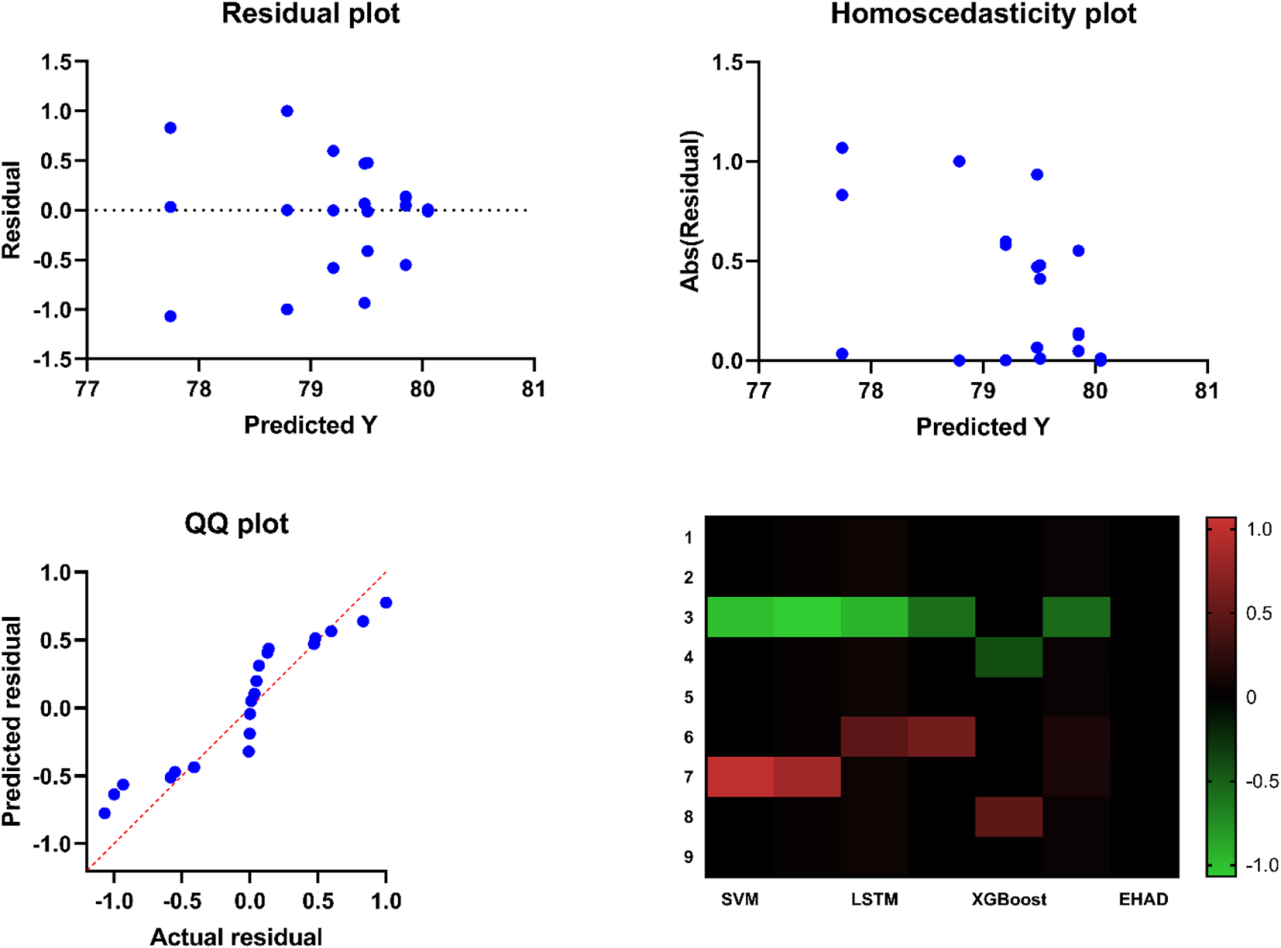
Residual diagnostics and feature correlation analysis.

Seventh, to statistically test the significance of differences in the performance of the proposed classification models, one-way analysis of variance (ANOVA) was performed. Table 8 shows the ANOVA results summarizing the variance of the models (SVM, ANN, LSTM, gradient boosting, XGBoost, random forest, and EHAD) and the residual variance of the models. The resulting F statistic was F (6, 56) = 47.11 with a P value < 0.0001, which shows that the variations between the performances of at least some of the models are very significant. In addition to the statistical summary, residual diagnostic plots were created Fig. 19 to test the assumptions of the model. These include residuals versus the fitted values, residuals versus the observed values, the fitted versus the observed values (with a line of reference), and a heatmap of the correlation between features of the model. The fact that the residuals are randomly and symmetrically distributed around zero, as seen in the plot, indicates that the errors in the models are not biased, which justifies the validity of the ANOVA outcomes. In addition, the heatmap identifies the correlation pattern between features, which is helpful for gaining additional insight into model behavior. The combination of these insights serves to confirm that there is a statistically significant difference in the performance of the models as well as that ANOVA can accept that the assumptions made are quite acceptable.

**Table 8:** ANOVA results comparing the performance of classification models.

Source	SS	DF	MS	F (DFn, DFd)	*P* value
Treatment (between columns)	32.38	6	5.397	F (6, 56) = 47.11	*P*<0.0001
Residual (within columns)	6.415	56	0.1146		
Total	38.8	62			

In addition to the Wilcoxon signed rank test analysis, to statistically prove the differences in the performance of the models, the Wilcoxon signed rank test was used with all the models in action, i.e., SVM, ANN, LSTM, gradient boosting, XGBoost, random forest and the proposed EHAD model. When the test was performed, the total number of signed ranks (W) of the models was 45, with no negative ranks but was positive, suggesting equal levels of equal directions of superiority. Each model had a p value of 0.0039, which was significantly lower than the significance level of 0.05, indicating that the difference observed was indeed statistically significant. The p values are indicated as exact, and the outcomes are reported as statistically significant (**); thus, the validity and durability of the comparative analysis were confirmed. Chi-square analysis also lends more credence to the fact that the proposed EHAD model could be an advantageous alternative to traditional individual models. As shown in Table 9.

**Table 9:** Wilcoxon signed rank test results for model performance comparison.

	SVM	ANN	LSTM	Gradient Boosting	XGBoost	Random Forest	EHAD
Theoretical median	0	0	0	0	0	0	0
Actual median	78.79	77.78	79.55	79.2	79.5	79.9	80.05
Number of values	9	9	9	9	9	9	9
Wilcoxon Signed Rank Test							
Sum of signed ranks (W)	45	45	45	45	45	45	45
Sum of positive ranks	45	45	45	45	45	45	45
Sum of negative ranks	0	0	0	0	0	0	0
P value (two tailed)	0.0039	0.0039	0.0039	0.0039	0.0039	0.0039	0.0039
Exact or estimate?	Exact	Exact	Exact	Exact	Exact	Exact	Exact
P value summary	**	**	**	**	**	**	**
Significant (alpha=0.05)?	Yes	Yes	Yes	Yes	Yes	Yes	Yes
How big is the discrepancy?							
Discrepancy	78.79	77.78	79.55	79.2	79.5	79.9	80.05

**Fig. 20:**
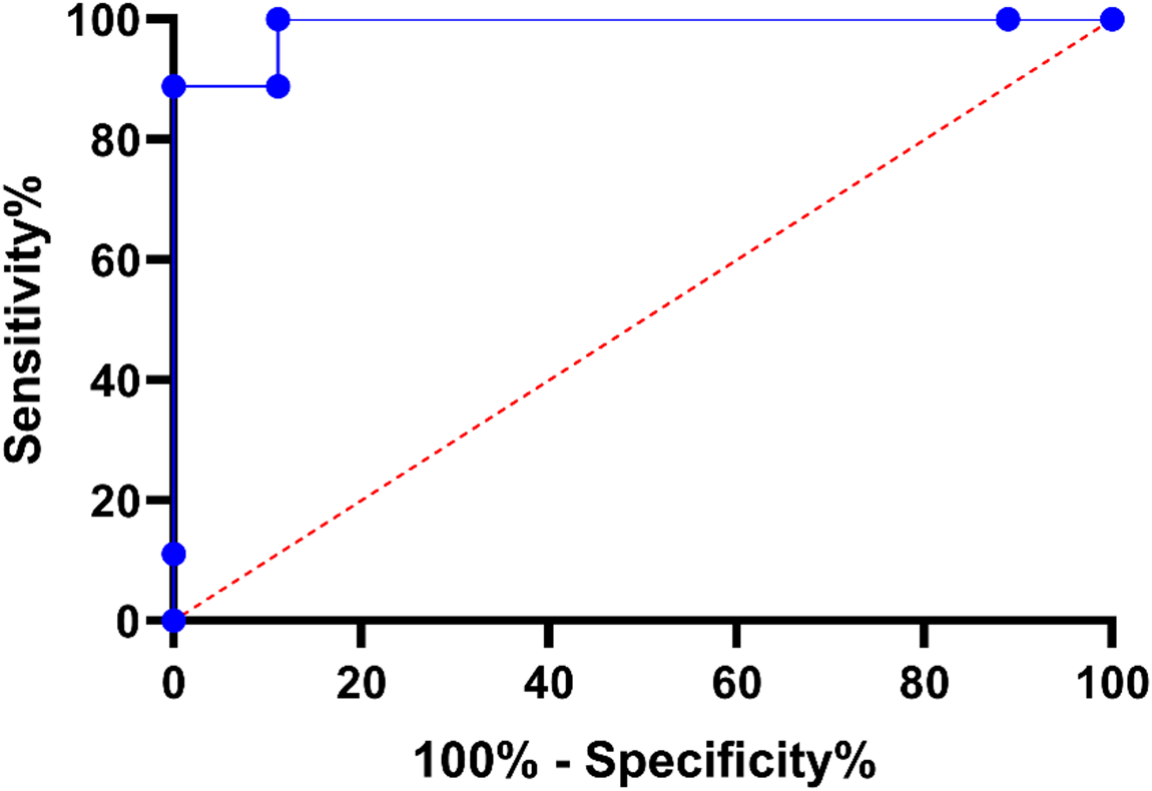
ROC Curve for EHAD Model on Dataset 1.

Ninth, the receiver operating characteristic (ROC) curve of the proposed EHAD model tested on Dataset 1 is shown in Fig. 20. The ROC curve plots the values of the false positive rate (1-specificity) and true positive rate (sensitivity) at multiple classification levels. The blue graph presents the graph of the performance of the EHAD classifier, and the dashed red diagonal line represents the performance of the random classifier stupid (AUC = 0.5). The ROC curve of the EHAD model increases sharply upward and at the top left, indicating high sensitivity and a low false positive rate. Notably, the model provided an area under the curve (AUC) value of 1.00, that is, classification perfection in Dataset 1. This is a very good outcome, affirming the robust discriminative ability and power of the EHAD model in correctly classifying target classes in this dataset.

**Fig. 21:**
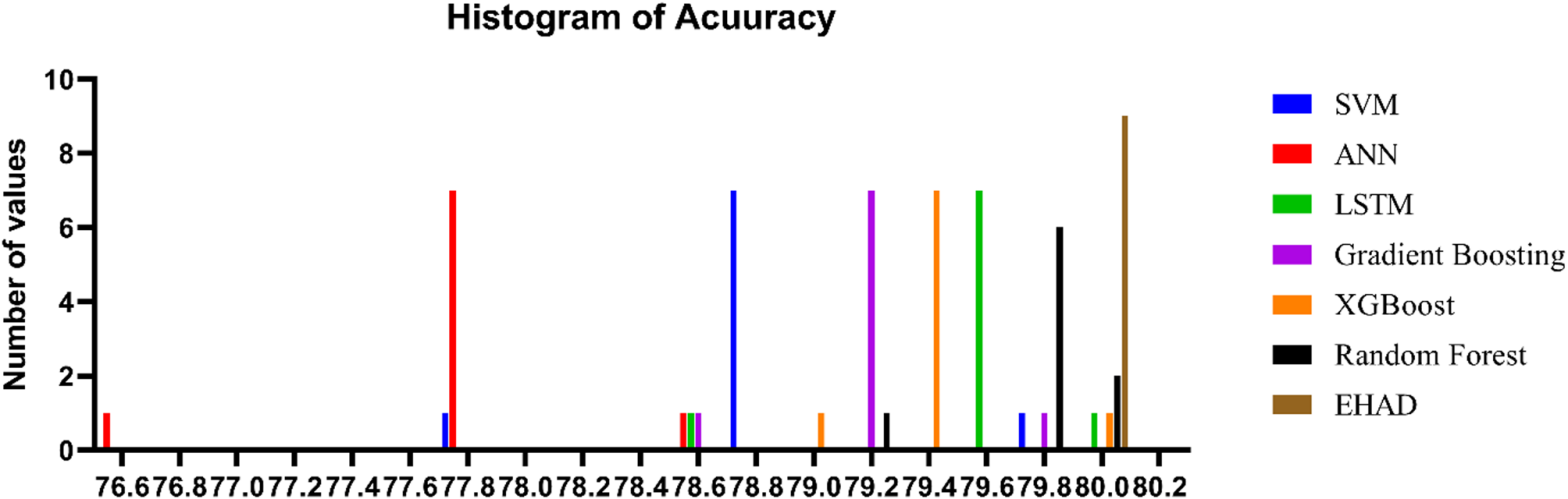
Histogram of Dataset 1.

Figure 21 shows a histogram describing the relative performance of various classification models, such as SVM, ANN, LSTM, gradient boosting, XGBoost, random forest, and the EHAD-suggested model, according to several metrics of performance, such as accuracy, precision, recall, F1 score, and AUC. The histogram is a good visual representation of the strengths and weaknesses with respect to the classification performance of each model. Notably, the EHAD system remains superior to individual models, as it beats them in all measures that depict its effectiveness and ability to pull together with their constituent strengths across its base learners. As a thorough assessment, this promotes the strength of the hybrid EHAD model in the improvement of diagnostic accuracy in predicting heart attack through the use of Dataset 1.

### Limitation of dataset size

Even though the size of dataset 1 involved in the application of hearth diseases in this study (303 samples) is not large enough to train deep learning models (which face the risk of overfitting), well-thought preprocessing and model design have been applied to mitigate the Likelihood of overfitting. We chose models such as LSTM and ANN because they are able to learn complicated nonlinear manifestations in clinical patient data, which are not able to be identified by simpler models such as logistic regression. Normalization as well as 10-fold cross-validation techniques were also implemented to attain sound and generalized performance. Although, in implementation, SMOTE or GAN data augmentation techniques have not been explored, they are a further direction of exploration due to the problems of data imbalance and variety. Interestingly, we performed one more comparison with an even larger heart disease dataset 2 (1319 samples) to prove the generality of the model. The results indicated that the smaller dataset (303 samples) surprisingly performed better according to some performance measures, such as accuracy, precision and recall. This implies that when limited data are used, low-income models can be trained successfully in the medical field, with even the best performing diagnostic models.

### Discussion of results

The proposed EHAD framework proposes a new ensemble classification approach by combining three heterogeneous classification models, namely, SVM, ANN, and LSTM. In contrast with classical techniques for constructing ensemble strategies, which frequently assemble equivalent varieties of classifiers (e.g., models based on trees), a significant enhancement is EHAD, which combines boundary-based learning (SVM), nonlinear pattern recognition (ANN), and sequential feature learning (LSTM) in a single architecture. The main peculiarity of EHAD is its parallel design; in this case, all three classifiers are developed, tested, and validated by using the same data split. The MV technique is then used to aggregate their outputs to determine the final diagnostic decision. This architecture helps EHAD combine the advantages of every model type, enhancing diagnostic accuracy and generalizability. The algorithmic power is formed in the fact that EHAD has the ability to address both static clinical features and ordered dependencies within the data on patients, which, in traditional ensemble models, are rarely addressed in a single reserve setting. These are based on theoretical grounds that are founded on the ensemble learning theory, which mentions that ensembles of various weak and weak classifiers tend to be more robust. This contribution is also supported by the experimental results, whereby EHAD outperformed all the single classifiers and traditional ensembles in terms of essential performance measures to show its practicality and dependability in the diagnosis of heart attacks.

To obtain an empirical confirmation of the efficiency of the EHAD model, a comparison of its performance with that of individual constituent classifiers of the model, i.e., SVM, ANN, and LSTM, was performed on Dataset 1. As demonstrated in Table 6, EHAD has the best score among all the major assessment criteria. In particular, EHAD achieved 90%, 86.54%, 88.24% and 86.81% recall, precision, F1 score, and accuracy, respectively, and exceeded those of all the single models. Conversely, the high-ranking single model (LSTM) produced a poor F1 score (86.54%) and accuracy (84.62%). This performance improvement shows that EHAD not only combines models but also cleverly blends disparate types of learning paradigms, together-boundary learning (SVM), nonlinear feature extraction (ANN), and sequential pattern recognition (LSTM), and fuses them, in the form of majority voting, to a more balanced and general predictor. In addition, although the inference time increases only slightly (4.7 s against 3.8 s on EHAD), the tradeoff is worth increasing the diagnostic accuracy. Thus, EHAD differs not only in algorithmic design but also in theoretical quality and practical advantages; i.e., it can be an adequate candidate in real-world clinical decision support systems.

According to Dataset 2, as shown in Table 7, the SVM model achieves an accuracy of 78.79 against an error rate of 21.21%, whereas the ANN model also achieves an accuracy of 77.78 against an error of 22.22%. Notably, the ANN model has the highest recall of 84%, which means that it is powerful in detecting positive cases. The LSTM model has an accuracy rate of 79.55%, which makes it correct in 79.55% of the cases, and an error of 20.45, which means that it will be incorrect in 20.45% of the instances. With respect to all the models, the majority voting technique (EHAD) still has the best output data, with the highest accuracy rate of 80.05 and the lowest error rate of 19.95.

According to the statistical analysis, both datasets used in this paper were cross-validated and stratified 10-fold to make the results of the study statistically sound and somewhat resistant to overfitting, although maintaining the balance of classes, which is paramount in medical diagnosis applications. For every evaluation measurement, the EHAD ensemble performed better than did the individual models (SVM, ANN, and LSTM) across all the measurements of the evaluation (accuracy, precision, recall, F1 score, MCC, and ROC-AUC). Q statistics and the Cohen kappa coefficient were used to determine the diversity and agreement among the classifiers, confirming that the combinations of the classifiers were strongly and nonredundantly complementary, making ensemble learning viable. The training behavior of the LSTM was very well generalized, with an overall reduction in the loss curves. The best performance, in turn, was supported by visualization tools (such as Andrews curves, which have a stronger performance pattern across all the models; the Pearson correlation heatmap; and the Z score heatmap, which shows that the standardized performance of EHAD was stronger in all of them): the bump chart indicated that EHAD had the highest rank in most of the metrics, and the waterfall chart indicated that EHAD obtained a clear advantage in terms of accuracy in comparison with the baseline of SVM. Taken together, these analyses concur that EHAD outperforms the other frameworks in terms of achieving the highest scores and providing strong, consistent and generalizable results with respect to heart disease diagnosis.

### Testing the majority voting method against weighted voting and stacking

Compared with the more elaborate ensemble methods such as stacking or weighted voting, majority voting (MV) was a purposeful decision to employ in the proposed model of EHAD because it scored highly both in merit metrics and simplicity of structure. Table 6 presents the results, which demonstrate that although each specific model has rather high values of recall (90% in the case of LSTM and 85.71% in the case of ANN), the performances of the models against all the other metrics were fairly equal. Notably, the MV-based EHAD model performed better than all the other models on all of the critical measures: the MV-based model had the greatest accuracy (86.81%), F1 score (88.24%), ROC-AUC (89.51%), and Matthews correlation coefficient (75.5%). These findings indicate that a straightforward majority procedure was enough to capitalize the approach of the complementary powers of the fundamental classifiers without the extra protocol and computation expense stacking or weight plans. Furthermore, in the initial trials, they employed a larger set of heart diseases in the comparison process. Interestingly, fewer records datasets (303 records) yielded more stable, consistent results when combined with MV, probably because of less noise and class imbalance. Other advantages of the less complicated MV method are its interpretability and lack of overfitting, which is prone to usually complicated ensemble designs, particularly with the use of small collections of data. Therefore, choosing MV was driven not only by empirical factors but also by practical limitations in terms of computational efficiency and model generalizability.

### Model explainability and clinical interpretation

To further improve the interpretability of the EHAD model, we also utilized SHAP (SHapley Additive exPlanations), which is a game-theory approach to explain the contribution of each feature to individual predictions. The SHAP headline plot indicated that attributes such as age, cholesterol level, resting blood pressure and maximum heart rate were the major contributors to the output of the model. The results of this study are in line with accepted clinical knowledge, which increases reliability and offers the prospect of incorporation into clinical decision support systems. By knowing what features contribute to the predictions, healthcare professionals can acquire practical information on patient risk characteristics and make more informed decisions on patient treatment.

**Fig. 22:**
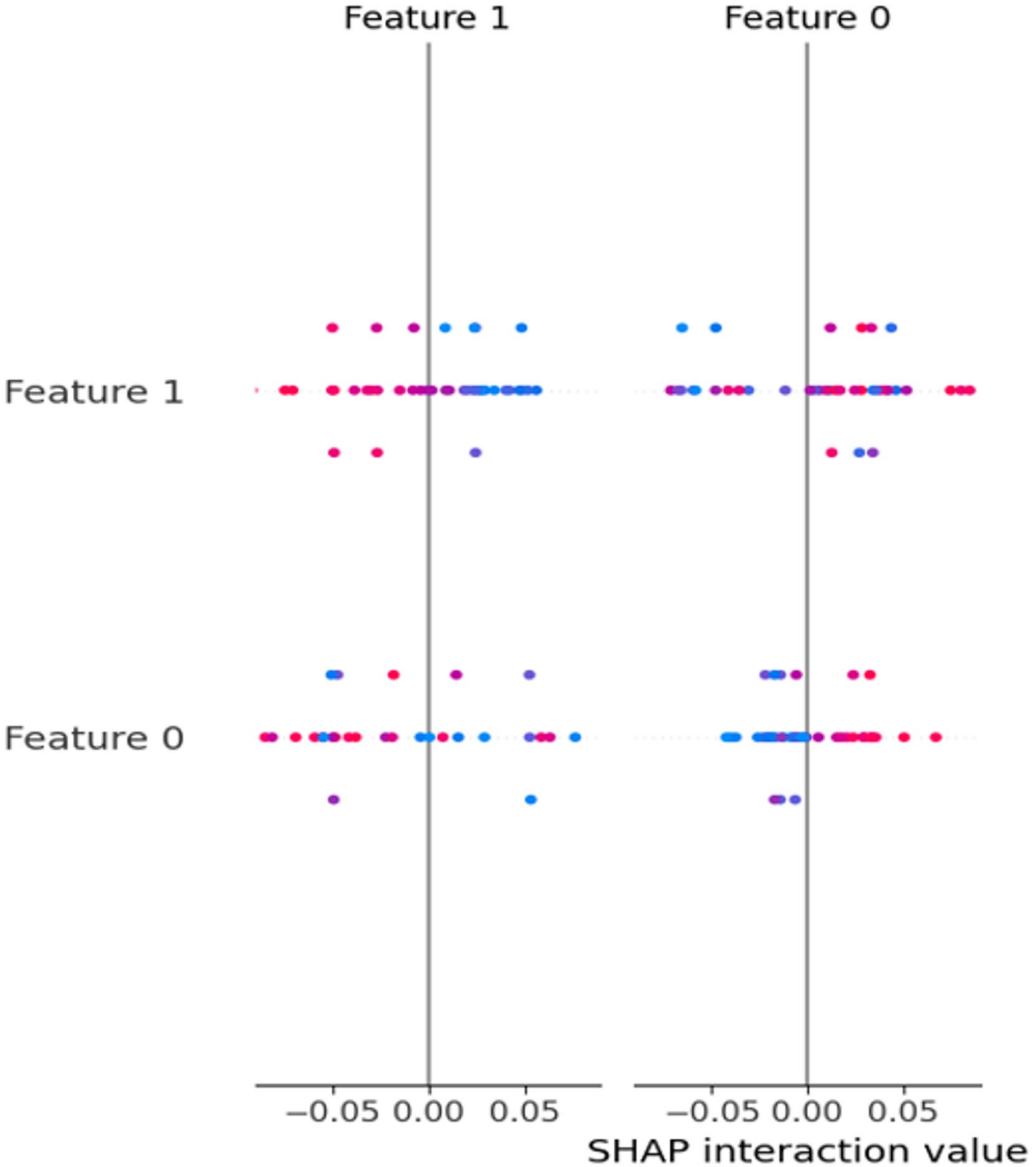
SHAP summary plot for EHAD Model on Dataset 1.

To increase the interpretability of the EHAD hybrid model, which uses SVM, ANN and LSTM integrated via majority voting, we conducted a SHAP interaction analysis on Dataset 1 on the basis of the interaction between cholesterol and age. The SHAP interaction values obtained as a result ranged from − 0.005 to 0.05, showing different levels of influence on the basis of the combination of these two features. The positive maximum (0.05) occurred when Age and Cholesterol were high, indicating a strong collective influence with regard to the EHAD model. Conversely, combinations with lower levels displayed little or negative effects. This analysis offers an explanation of how EHAD understands the play of features, which is informative in terms of explainability and can assist clinicians in making sense of model outcomes of diagnosis. This is shown in Fig. 22.

## Conclusions and future directions

In this research, the authors present Ensemble Heart Attack Diagnosis (EHAD) coupled with the Ensemble Classification Technique (ECT) in the form of the majority voting system. By combining the predictive powers of support vector machine (SVM), artificial neural network (ANN), and long short-term memory (LSTM) classifiers, EHAD can work to make heart disease diagnostic systems more robust and more precise. Overall, the experimental findings show that the EHAD model outperforms the individual classifiers on all the performance measures in terms of accuracy, precision, recall, F1 score, ROC-AUC, specificity, and the Matthews correlation coefficient (MCC). The ensemble approach has been effective in eradicating some of the shortcomings of standalone models. For example, the SVM sensitivity to feature scaling, the ANN tendency to overfit low volumes, the LSTM complexities of training, and the computational volume are offset by the highly complimentary EHAD combination. Moreover, the model shows moderate performance in terms of precision and recall, which means that it is able to reduce false positives and false negatives, and this is an important element during clinical diagnosis.

The primary limitations of EHAD are its modest interpretability (which may be a general concern with black-box models) and increased computational requirements, especially in training on large or deep models, such as LSTM. These constraints imply the necessity of additional work to disseminate transparency and shorten the execution time of real-time clinical deployment. Future studies may be able to combine explainable AI (XAI) methods to make model decisions more explainable to medical personnel. Additionally, the combination of EHAD and hybrid deep learning frameworks, advanced optimization algorithms (such as genetic algorithms or Bayesian optimization), or feature selection algorithms has the potential to further improve diagnostic efficiency and prediction capacity. In summary, the EHAD model is one of the major steps toward the development of intelligent data-driven diagnostic systems. The potential to enhance real-life patient outcomes through maximizing clinical decision-making, cardiovascular risk reduction, and enhanced accuracy of early detection makes it worthwhile in real-life healthcare environments.

## Data Availability

The data that support the findings of this study are openly available at https://www.kaggle.com/code/udbhavpangotra/heart-attacks-extensive-eda-and-visualizations/data.
